# Ferroptosis–Senescence Crosstalk in Sepsis-Associated Acute Lung Injury: Mechanisms and Therapeutic Opportunities

**DOI:** 10.3390/biomedicines14081869

**Published:** 2026-08-21

**Authors:** Renwei Luo, Qingyun Chen, Jiaxing Wang, Zhihao Nie, Lingxuan Dan, Songping Xie

**Affiliations:** Department of Thoracic Surgery, Renmin Hospital of Wuhan University, Wuhan 430060, China; 2023203020029@whu.edu.cn (R.L.); 2024283020138@whu.edu.cn (Q.C.);

**Keywords:** sepsis-associated acute lung injury (SALI), ferroptosis, cellular senescence, oxidative stress, redox homeostasis

## Abstract

Sepsis-associated acute lung injury (SALI) is characterized by disruption of the alveolar–capillary barrier, uncontrolled inflammation, oxidative stress, and impaired tissue repair. Ferroptosis and cellular senescence have emerged as potentially interacting stress-response programs that may jointly shape the progression of septic lung injury. Ferroptosis promotes epithelial and endothelial damage through iron-dependent lipid peroxidation, glutathione depletion, and impaired GPX4-mediated lipid repair. In parallel, senescence-associated remodeling may contribute to persistent cell-cycle arrest, senescence-associated secretory phenotype (SASP) production, endothelial dysfunction, and defective regenerative capacity. This review summarizes current evidence on the molecular and cellular crosstalk between ferroptosis and cellular senescence in SALI. Candidate regulatory intersections include context-dependent mitochondrial dysfunction, reactive oxygen species accumulation, iron dyshomeostasis, metabolic reprogramming, lysosomal dysfunction, DNA-damage responses, and stress-responsive pathways involving p53, NRF2, ATF4, STAT3, and FOXO1. Direct SALI evidence is currently strongest for ferroptosis-induced senescence-associated remodeling in pulmonary endothelial cells, whereas senescence-associated ferroptosis resistance is supported mainly by non-pulmonary models. Likewise, SASP-mediated paracrine ferroptosis in neighboring pulmonary cells remains insufficiently validated. We therefore propose an evidence-informed, temporally and cell-type-dependent ferroptosis–senescence framework in SALI, in which acute senescence-associated responses may coexist with ferroptotic injury, whereas persistent senescence-associated remodeling may contribute to defective repair and microenvironmental injury amplification. Targeting this axis through ferroptosis inhibition, restoration of endogenous antioxidant defenses, senotherapeutic modulation, and regenerative strategies may offer stage-informed therapeutic opportunities. Further time-resolved and cell-specific studies are required to define causal relationships and clinically actionable therapeutic windows.

## 1. Introduction

Sepsis is defined as life-threatening organ dysfunction caused by a dysregulated host response to infection [1]. It remains a major global health burden [2]. The lung is particularly susceptible to sepsis-related injury, resulting in sepsis-associated acute lung injury (SALI); severe clinical pulmonary involvement may manifest as sepsis-associated acute respiratory distress syndrome (SA-ARDS) [3,4]. SALI is characterized by inflammatory injury to the alveolar–capillary unit, including pulmonary endothelial activation, epithelial barrier disruption, increased vascular permeability, protein-rich pulmonary edema, and impaired gas exchange [4,5,6]. In severe cases, these abnormalities may culminate in diffuse alveolar damage and profound hypoxemic respiratory failure [3,7,8,9]. Despite advances in early antimicrobial therapy, source control, hemodynamic optimization, and lung-protective ventilation, treatment remains largely supportive, and no pharmacological strategy directly targeting the molecular injury–repair programs of SALI has yet demonstrated consistent clinical benefit [7,10]. This unmet need reflects the marked temporal and cellular heterogeneity of septic lung injury, in which early stress-adaptive responses may transition to maladaptive cell death, persistent cellular dysfunction, and defective tissue repair [11]. Defining the regulatory networks that govern these transitions is therefore essential for the development of more precise and stage-specific therapeutic strategies for SALI.

Against this background, dysregulated cell-fate control has emerged as a key component of SALI pathogenesis [12,13]. Multiple regulated cell-death programs, including apoptosis, pyroptosis, and necroptosis, contribute to septic lung injury, while autophagy modulates cellular stress responses and death susceptibility [13,14]. This review focuses on ferroptosis and cellular senescence because they define a distinct injury–repair interface: ferroptosis drives acute iron- and lipid-peroxidation-dependent epithelial and endothelial injury, whereas senescence is a persistent, non-lethal state associated with proliferative arrest, secretory remodeling, and impaired regeneration. Their shared redox, iron-handling, mitochondrial, lysosomal, and metabolic regulators, together with direct evidence linking pulmonary endothelial ferroptosis to senescence in SALI, provide the mechanistic basis for this focused scope [15,16]. This selection does not imply that ferroptosis predominates over other death pathways, but enables detailed analysis of the transition from acute barrier injury to persistent dysfunction and defective repair. Ferroptosis is an iron-dependent form of regulated cell death driven by the accumulation of membrane lipid peroxides [17,18,19]. It is closely linked to impaired lipid-peroxide detoxification, iron dyshomeostasis, redox imbalance, and lipid-metabolic remodeling [17,18,20]. Current evidence, derived mainly from experimental sepsis models, indicates that ferroptotic stress affects alveolar epithelial and pulmonary microvascular endothelial cells, while inflammatory immune signals can further modulate this process [21,22,23,24]. By impairing epithelial viability and endothelial barrier integrity, ferroptosis may increase alveolar–capillary permeability, amplify inflammatory signaling, and aggravate microvascular dysfunction [21,23,24]. Notably, its major upstream drivers, including oxidative stress, mitochondrial dysfunction, iron dyshomeostasis, and inflammation-associated metabolic reprogramming, also overlap with pathways implicated in cellular senescence, providing a mechanistic bridge to defective tissue repair [15,20]. At the molecular level, p53 links DNA-damage signaling and p21-dependent cell-cycle arrest to context-dependent regulation of SLC7A11, whereas NRF2 and the GSH–GPX4/FSP1 systems determine resistance to lipid peroxidation [25,26,27,28,29]. FOXO1-mediated mitochondrial quality control, together with lysosomal iron handling and ferritinophagy, may further modify ferroptosis susceptibility and senescence-associated phenotypes [30,31,32]. In addition, SASP-related NF-κB and STAT3 signaling may propagate oxidative and inflammatory stress to neighboring pulmonary cells, although several of these reverse and paracrine connections remain incompletely validated in SALI [16,33,34,35,36,37].

Cellular senescence represents another stress-responsive program that may shape the transition from acute injury to defective repair in SALI [38,39,40]. Classically, senescence is characterized by durable cell-cycle arrest accompanied by metabolic remodeling, persistent DNA-damage responses, and the senescence-associated secretory phenotype (SASP) [38,41]. In septic lung injury, oxidative stress, inflammatory signaling, and mitochondrial dysfunction may induce early senescence-associated phenotypes in alveolar epithelial and pulmonary endothelial cells [38,39,40]. These cells can release SASP mediators that sustain inflammation, alter immune-cell recruitment, and remodel the extracellular matrix [38,39,41]. Although transient activation of senescence-associated programs may limit proliferation of damaged cells and facilitate tissue adaptation, their persistence can impair epithelial regeneration, compromise endothelial repair, and favor maladaptive remodeling [40,42,43]. Accordingly, defining how senescence interfaces with ferroptosis across cell types and injury stages may clarify the shift from adaptation to failed repair in SALI.

Ferroptosis and cellular senescence may constitute a context-dependent stress-response network rather than isolated processes in SALI [15,44]. Both can be initiated by oxidative stress, mitochondrial dysfunction, iron dyshomeostasis, and inflammation-associated metabolic remodeling [15,16]. Available sepsis-specific evidence indicates that ferroptotic injury in pulmonary vascular endothelial cells can promote a senescence phenotype and worsen lung injury [16]. Conversely, senescence-associated alterations in lysosomal iron handling, lipid metabolism, and antioxidant defenses may modify cellular susceptibility to ferroptosis; however, whether this reverse regulation operates in SALI remains largely unresolved [30,31].

Previous reviews have largely addressed ferroptosis in SALI or ALI, cellular senescence in acute lung injury or sepsis, or ferroptosis–senescence interactions across heterogeneous diseases [12,14,15,23,38,39,44,45]. However, it remains unclear which proposed connections are directly supported in SALI, how they differ among AT2 epithelial, pulmonary endothelial, and immune-cell compartments and across injury stages, and whether they reflect transient stress responses or persistent senescence.

This review addresses these gaps by applying a SALI-specific evidence hierarchy and organizing the evidence according to cell type, injury stage, and cell-autonomous versus paracrine effects. We propose an evidence-informed spatiotemporal framework linking acute epithelial–endothelial injury to defective resolution while identifying unvalidated mechanisms. We also evaluate candidate circulating and bronchoalveolar biomarkers, stage-adapted therapies, translational barriers, and feasible experimental validation strategies.

## 2. Ferroptosis: Molecular Basis and Its Role in Acute Lung Injury

### 2.1. Molecular Basis and Core Execution Mechanisms

Ferroptosis is a distinct form of regulated cell death initiated by oxidative perturbations and driven by the iron-dependent accumulation of phospholipid hydroperoxides under the constitutive control of GPX4 [17,18,19,46,47]. Its execution results from the convergence of disturbed iron handling, enrichment of oxidizable polyunsaturated fatty acid-containing phospholipids (PUFA-PLs), and failure of lipid-peroxide detoxification systems [17,18,20,46,48]. During sepsis, inflammatory and metabolic stress can perturb redox and iron homeostasis [20,23,24]. In experimental models, these perturbations increase ferroptosis susceptibility in pulmonary endothelial and epithelial cells [21,22,24]. Mechanistically, ferroptosis depends on the size and subcellular distribution of the redox-active labile Fe^2+^ pool rather than on total cellular iron alone. Fe^2+^ promotes Fenton-type radical generation and can support lipoxygenase-mediated oxidation of PUFA-containing phospholipids, thereby initiating and propagating membrane lipid-peroxide chains. Accordingly, transferrin/TFRC-mediated uptake, ferritin storage and ferritinophagy, and SLC40A1-dependent export can modify ferroptosis susceptibility by controlling the availability of catalytic iron [20,30,31,46,49,50,51]. Among these defenses, the cystine/glutathione/GPX4 system is central [17,18,46]. System Xc^−^, composed of SLC7A11 and SLC3A2, imports cystine to support glutathione synthesis, while GPX4 uses glutathione to reduce phospholipid hydroperoxides [17,52,53]. In parallel, ACSL4 and LPCAT3 enrich membrane phosphatidylethanolamines with arachidonic acid (AA; 20:4) and adrenic acid (AdA; 22:4) [54,55]. Their multiple double bonds create bis-allylic C–H sites that are readily attacked by radicals, making these phospholipids particularly susceptible to iron-dependent peroxidation and propagation of ferroptotic membrane damage [54,55]. Ferroptosis therefore occurs when lipid-peroxide generation exceeds endogenous detoxification capacity. Importantly, mitochondria are not an obligatory execution platform for all forms of ferroptosis. In non-pulmonary experimental systems, mitochondrial tricarboxylic acid cycle activity, electron-transport-chain function, and glutaminolysis are required for cysteine-deprivation-induced ferroptosis, whereas mitochondria are dispensable when ferroptosis is triggered by direct GPX4 inhibition [56]. More broadly, distinct ferroptotic stimuli depend on different upstream metabolic and lipid-regulatory factors while converging on lethal phospholipid-peroxide accumulation at cellular membranes [46,57].

Iron-dependent ferroptosis in the alveolar epithelium is further shaped by the balance among iron uptake, ferritin storage, iron export, and intracellular iron mobilization [20,24,46]. In pulmonary epithelial cells, NCOA4-dependent ferritinophagy can release ferritin-bound Fe^2+^, whereas SFXN1-associated mitochondrial iron transport increases mitochondrial iron loading and oxidative stress. In a CLP model, epithelial YAP1 deficiency enhanced NCOA4–FTH1 interaction, mitochondrial iron accumulation, ROS generation, and ferroptotic lung injury [58]. Conversely, recent evidence from LPS-induced ALI and primary AT2 cells indicates that SLC38A1 promotes HSP90/HSC70/LAMP2A-dependent chaperone-mediated autophagic degradation of DMT1, thereby limiting iron accumulation, mitochondrial dysfunction, and AT2-cell ferroptosis [59]. These findings suggest that both ferritin iron release and DMT1-dependent iron uptake influence the labile and mitochondrial iron pools in the injured alveolar epithelium.

Consistent with this framework, multiple ferroptosis-associated alterations have been reported in CLP- and LPS-based models of septic lung injury, including increased iron accumulation and lipid peroxidation, reduced glutathione availability and GPX4 expression, and, in selected ultrastructural studies, condensed mitochondria with reduced cristae [14,23,24,60]. These features should be interpreted collectively, as no single marker is sufficient to establish ferroptosis in vivo [17,18,46]. In LPS-induced acute lung injury, ferrostatin-1 reduced histological injury, pulmonary lipid peroxidation, and inflammatory mediator release while restoring SLC7A11 and GPX4 expression [61]. Similarly, interventions that preserve antioxidant capacity or iron homeostasis alleviate ferroptosis-associated injury in CLP models [45,62,63,64]. Collectively, these preclinical findings support ferroptosis as a contributory mechanism of epithelial and endothelial barrier failure in SALI, while highlighting that its relative importance may vary across models, cell types, and disease stages. This evidence provides a rationale for examining the upstream regulatory networks that determine ferroptosis susceptibility in the septic lung.

### 2.2. Regulatory Networks and Intercellular Amplification of Ferroptosis

Building on the core execution machinery outlined above, ferroptosis in SALI is further regulated by upstream networks that extend beyond individual cells. Within the inflammatory lung microenvironment, immune-to-parenchymal communication, transcriptional and post-transcriptional regulation, metabolic and epigenetic reprogramming, and mitochondrial quality control collectively modify iron handling, membrane lipid vulnerability, and lipid-peroxide detoxification capacity [21,22,32,50,51,65,66,67]. These regulatory layers act in a cell-type- and stage-dependent manner, such that pulmonary endothelial and alveolar epithelial cells may integrate distinct inflammatory and metabolic cues and exhibit distinct susceptibilities to ferroptotic injury [21,22,50,51]. The following sections summarize how these interconnected mechanisms amplify or restrain ferroptotic injury and thereby contribute to alveolar–capillary barrier failure in SALI. These mechanisms converge on shared ferroptotic effector systems but operate through distinct upstream pathways in alveolar epithelial and pulmonary endothelial cells. Mechanistically, failure of GPX4- and FSP1-dependent lipid repair allows oxidized phospholipids to accumulate in epithelial and endothelial membranes, compromising membrane integrity and causing ferroptotic loss of barrier-forming cells. NINJ1-associated membrane rupture and the release of DAMPs and oxidized lipids can further amplify inflammatory injury, thereby increasing alveolar–capillary permeability and protein-rich edema [49,60,64].

At the cell-intrinsic level, transcriptional and post-transcriptional circuits shape susceptibility to ferroptotic injury in SALI. NRF2 is a central anti-ferroptotic regulator that coordinates cystine utilization, glutathione synthesis, iron sequestration, and antioxidant responses [25,26]. In experimental sepsis-induced lung injury, the RNA-binding protein AUF1 counteracts ferroptosis by differentially regulating the stability of NRF2 and ATF3 transcripts; its degradation by FBXW7 weakens this protective program [25]. The p53–SLC7A11 axis represents an additional stress-responsive checkpoint, although the effect of p53 on ferroptosis is context-dependent [27,28,29]. STAT3 activation has also been associated with ferroptosis in sepsis-associated ARDS, whereas ER stress-linked PERK–ATF4 signaling may further modulate ferroptosis susceptibility [33,34,68]. Together, these pathways integrate oxidative, inflammatory, and metabolic stress signals to determine iron handling, glutathione availability, mitochondrial redox balance, and ferroptosis susceptibility in a cell- and stimulus-dependent manner.

Immune-to-parenchymal communication provides a direct route through which ferroptotic stress is amplified in SALI. Macrophage-derived extracellular vesicles deliver guanylate-binding protein 2 (GBP2) to pulmonary microvascular endothelial cells, where GBP2 interacts with OTUD5 and promotes GPX4 ubiquitination and degradation, thereby enhancing endothelial ferroptosis and impairing vascular barrier integrity [21]. In contrast, NETs act predominantly on the alveolar epithelial compartment. NET exposure promotes METTL3-dependent m6A regulation of *GPX4* mRNA, reduces GPX4 expression and glutathione-dependent lipid-peroxide detoxification, and increases epithelial ferroptotic injury [22]. These findings demonstrate that distinct immune-cell-derived signals converge on GPX4-dependent defense in different cellular compartments of the alveolar–capillary unit.

In alveolar epithelial cells, ferroptosis susceptibility is regulated not only by GPX4 abundance but also by coordinated changes in iron mobilization, mitochondrial redox homeostasis, membrane lipid composition, and glutathione synthesis [20,22,24,46,54,55,65,69,70]. NCOA4-dependent ferritinophagy and DMT1-dependent iron uptake alter the cytosolic and mitochondrial iron pools, whereas ACSL4 and LPCAT-family enzymes determine the abundance of oxidizable PUFA-containing phospholipids. These iron- and lipid-metabolic processes interact with the system Xc^−^–GSH–GPX4 pathway to determine whether oxidative stress is buffered or progresses to ferroptotic epithelial injury [58,59].

Metabolic reprogramming links the septic inflammatory response to ferroptosis through lactate-dependent epigenetic signaling. Enhanced glycolytic flux during sepsis increases lactate availability, which may alter chromatin accessibility and ferroptosis-related gene expression [65,67,70]. NETs promote METTL3-dependent m6A regulation of *GPX4* mRNA, thereby weakening GPX4-mediated lipid-peroxide detoxification [22]. A parallel pathway involves METTL4-mediated m6A modification of the 3′ untranslated region of *NFE2L2* mRNA and YTHDF2-dependent transcript degradation, which suppresses the NRF2–SLC7A11–GSH–GPX4 antioxidant program and increases Fe^2+^ and lipid ROS accumulation [69]. Lactate-dependent modifications converge on both lipid remodeling and glutathione metabolism. GPR81 activation promotes p300-dependent H3K18 lactylation at the *METTL3* promoter, followed by METTL3-mediated m6A modification and YTHDC1-dependent stabilization of *ACSL4* mRNA, thereby enhancing PUFA-PL remodeling and mitochondria-associated ferroptosis [65]. In parallel, PDK4-driven lactate accumulation promotes AARS1-dependent lactylation of LPCAT2, facilitates STAT1 activation and nuclear translocation, and represses *SLC7A11* transcription, resulting in reduced GSH synthesis and increased epithelial ferroptosis [70]. Thus, m6A and lactylation do not represent isolated epigenetic events; rather, they converge on ACSL4/LPCAT-dependent lipid remodeling and the NRF2–system Xc^−^–GSH–GPX4 defense network.

Importantly, mitochondrial involvement is not universally required for ferroptosis and varies according to the initiating stimulus and metabolic context. Evidence from non-pulmonary systems indicates that mitochondrial tricarboxylic acid cycle activity, electron-transport-chain function, and glutaminolysis contribute to cystine-deprivation-induced ferroptosis, whereas mitochondria are largely dispensable when ferroptosis is initiated by direct GPX4 inhibition [46,56,57]. Mitochondrial morphological alterations and oxidative dysfunction should therefore be interpreted as supportive or regulatory features rather than sufficient evidence of obligatory mitochondrial dependence.

Mitochondrial quality control is a context-dependent modifier of ferroptosis susceptibility in SALI. In pulmonary endothelial cells, DPEP1 silencing restores FOXO1 and ALDH1L2 expression, improves fatty-acid oxidation and mitochondrial homeostasis, and attenuates oxidative stress and ferroptosis [32]. This pathway is highlighted as a recent SALI-specific example of endothelial mitochondrial regulation, rather than because its evidence base exceeds that of the more extensively validated SLC7A11–GSH–GPX4, ACSL4, or NRF2 pathways; independent replication remains necessary [32]. More recent endothelial evidence indicates that the pathological interaction between FUNDC1 and GPX4 can promote mitophagic degradation of GPX4 and disrupt mitophagic flux; interruption of this interaction stabilizes GPX4 activity, preserves mitochondrial homeostasis, and reduces endothelial ferroptosis and vascular leakage [71]. Together, these findings link mitochondrial quality control to GPX4-dependent lipid repair. However, the FUNDC1–GPX4 mechanism has been demonstrated in pulmonary endothelial cells and should not be regarded as direct evidence of an equivalent mitophagy–GPX4 pathway in AT2 cells.

Accordingly, the DPEP1–FOXO1–ALDH1L2 and FUNDC1–GPX4 pathways should be considered experimentally supported, endothelial-specific mechanisms under defined SALI conditions, whereas their generalization to AT2 cells, other pulmonary cell populations, or all ferroptotic stimuli remains hypothetical. Whether mitochondrial quality control is necessary or sufficient for ferroptosis in each pulmonary compartment requires cell-specific genetic perturbation combined with distinct ferroptosis-inducing conditions.

Taken together, ferroptosis susceptibility in SALI is governed by an integrated but cell-specific iron–mitochondrial–lipid–glutathione network. In alveolar epithelial cells, ferritinophagy and DMT1-dependent iron uptake influence the labile and mitochondrial iron pools; m6A and lactylation regulate ACSL4/LPCAT-dependent PUFA-PL remodeling and the NRF2–system Xc^−^–GSH–GPX4 antioxidant system; and mitochondrial iron loading and metabolic dysfunction increase oxidative pressure. In pulmonary endothelial cells, extracellular vesicle cargo, histone lactylation, iron-transport dysregulation, and mitochondrial quality-control pathways similarly converge on GPX4 stability, lipid peroxidation, and barrier dysfunction. However, these mechanisms were established in separate experimental models, and no single AT2-specific SALI study has yet causally integrated ferritinophagy or mitophagy, mitochondrial iron loading, lactylation, m6A regulation, lipid remodeling, and glutathione metabolism. This framework should therefore be interpreted as an evidence-based integrative network rather than a fully validated linear signaling axis.

## 3. Cellular Senescence in Acute Lung Injury

### 3.1. Biological Features and Cellular Sources of Senescence in SALI

**Figure 1:**
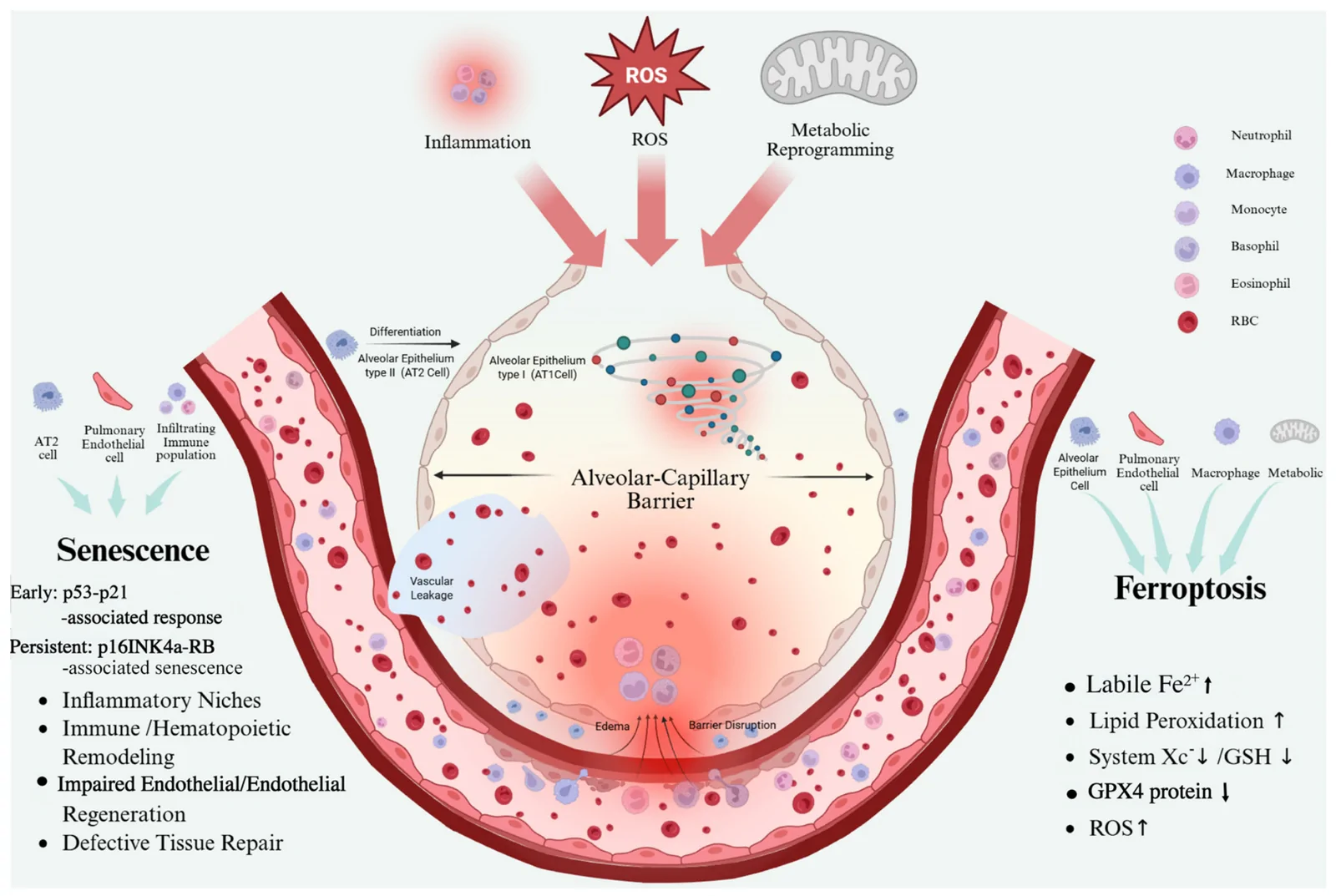
Cell-specific contributions of ferroptosis and senescence-associated remodeling to alveolar–capillary barrier failure in SALI. Sepsis-associated inflammation, oxidative stress, and metabolic reprogramming promote ferroptotic injury and senescence-associated responses in alveolar epithelial, pulmonary endothelial, and immune-cell compartments. Acute ferroptotic injury is characterized by increased labile iron and lipid peroxidation together with impaired antioxidant defense, whereas persistent senescence-associated remodeling may contribute to defective epithelial–endothelial regeneration, inflammatory persistence, and failed tissue repair. Arrows indicate the direction of the depicted cellular, pathological, and regulatory processes. Created in BioRender. Chen, Q. (2026) https://BioRender.com/77coc98.

Building on the ferroptosis-centered injury networks described above, cellular senescence represents a stress-responsive cell state that may contribute to the transition from acute injury to defective repair in SALI [38,39,72]. As illustrated in Figure 1, persistent inflammation, excessive ROS, mitochondrial stress, and metabolic reprogramming can converge to elicit senescence-associated programs within the alveolar–capillary unit [38,39,40,72]. Senescence is characterized by stable cell-cycle exit, extensive transcriptional and metabolic remodeling, and acquisition of a senescence-associated secretory phenotype (SASP), rather than by any single marker [41,73,74,75,76,77,78]. In the septic lung, activation of the p53–p21 and p16INK4a–RB pathways, together with persistent DNA-damage signaling and chromatin reorganization, may accompany this state. Senescence-associated phenotypes in SALI should be interpreted along a temporal continuum rather than as a strict p21-versus-p16 dichotomy. In experimental sepsis, pulmonary p21 expression is increased at 24 h after CLP, whereas p16 expression is reduced, supporting an early p21-dominant senescence-like response rather than fully established chronic senescence [72]. However, these findings were obtained primarily from whole-lung tissue and therefore do not resolve AT2 epithelial, endothelial, macrophage, or other cell-specific expression patterns. Persistent p16INK4a–RB activation, together with sustained cell-cycle arrest, persistent DNA-damage signaling, lamin B1 loss, increased senescence-associated β-galactosidase activity, and acquisition of a defined SASP, would provide stronger evidence of established cellular senescence [74,75]. Accordingly, throughout this review, “senescence-associated response” denotes an acute, potentially reversible stress-arrest phenotype without demonstrated persistence, whereas “established cellular senescence” requires durable growth arrest beyond the initiating insult and concordant multidimensional evidence. SASP mediators, including cytokines, chemokines, and matrix-remodeling factors, can reshape the alveolar–capillary microenvironment by sustaining inflammation, altering immune-cell recruitment, and limiting reparative responses [35,38].

Within the alveolar–capillary unit, senescence-associated phenotypes may arise in alveolar type II (AT2) epithelial cells, pulmonary endothelial cells, and selected immune-cell populations, although the strength of evidence differs across these compartments [38,39,40,42].

### 3.2. Functional Impact of Senescence on Lung Injury and Repair Failure

The functional consequences of senescence-associated remodeling extend beyond cell-cycle arrest and include impaired barrier maintenance and defective vascular repair [38,74]. Alveolar epithelial repair is also essential, because senescence-associated dysfunction of AT2 progenitors may restrict self-renewal and AT2-to-AT1 differentiation, although direct lineage-resolved evidence in SALI remains limited [40,42,79]. Within the alveolar–capillary unit, endothelial dysfunction is particularly consequential because pulmonary endothelial cells are required for both barrier integrity and regenerative recovery after septic injury [4,80,81]. In experimental sepsis models, endothelial ferroptosis can induce a senescence phenotype that is associated with reduced proliferative capacity, increased permeability, and aggravated lung injury [16]. However, the direct contribution of persistent endothelial senescence to barrier failure in SALI requires further clarification [16]. FoxM1 is not a senescence marker but a key regulator of endothelial cell-cycle re-entry and vascular repair [80,81]. In septic and aged lungs, inadequate FoxM1 induction impairs endothelial regeneration, whereas its reactivation promotes barrier restoration and resolution of inflammatory injury [80,81]. Thus, senescence-associated endothelial dysfunction and defective FoxM1-dependent repair may cooperate to prolong vascular leakage and delay alveolar–capillary recovery in SALI.

Beyond defective structural repair, senescence-associated remodeling may also perpetuate a maladaptive inflammatory niche in SALI [38,43,82]. Senescent epithelial and endothelial cells can generate a SASP composed of cytokines, chemokines, proteases, and matrix-modifying factors, thereby altering leukocyte recruitment and cell–cell communication within the alveolar–capillary unit [35,36,74]. When sustained, this secretory program may reinforce local oxidative and inflammatory stress, impair progenitor-cell function, and favor aberrant extracellular-matrix remodeling [36,42,43]. SASP activity may therefore link early injury responses to delayed resolution and a profibrotic repair trajectory [36,42,79]. However, the cellular composition, temporal persistence, and clinical relevance of the SASP in human SA-ARDS remain insufficiently defined [38,43]. Clarifying these dynamics will be essential to distinguish transient adaptive signaling from persistent senescence-associated pathology.

In addition to SASP-mediated niche remodeling, sepsis is associated with non-canonical immune-aging phenotypes that may contribute to sustained immune dysregulation [39,83,84,85,86,87]. In a CLP model, blockade of JAM-C reduced the accumulation of CXCR4^+^ aged neutrophils in the circulation and lungs and was accompanied by decreased pulmonary neutrophil infiltration, inflammatory mediator production, and histological injury [83]. These findings support a role for altered neutrophil aging, survival, and trafficking in acute septic lung inflammation. However, CXCR4 expression and prolonged neutrophil survival should not be interpreted as evidence of canonical cellular senescence, because canonical proliferative-arrest criteria are not directly applicable to terminally differentiated neutrophils, and a stable senescence-associated secretory program has not been demonstrated in this population [74,75].

Separately, preclinical and human studies indicate that sepsis can induce persistent alterations in the abundance, responsiveness, and myeloid differentiation of hematopoietic stem and progenitor cells (HSPCs) [84,85,86,87]. These studies support systemic hematopoietic and myeloid reprogramming during or after sepsis, but they do not demonstrate that HSPC remodeling directly induces pulmonary cellular senescence, ferroptosis, or failed alveolar–capillary repair. CXCR4^+^ aged neutrophil accumulation and HSPC reprogramming should therefore be regarded as parallel manifestations of sepsis-associated immune dysregulation rather than components of an established immunosenescence–parenchymal ferroptosis cascade in SALI.

Direct immune-to-parenchymal ferroptosis pathways have nevertheless been identified in SALI. NETs promote METTL3-dependent m6A regulation of *GPX4* mRNA and induce ferroptosis in alveolar epithelial cells [22]. In parallel, macrophage-derived extracellular vesicles deliver GBP2 to pulmonary endothelial cells, where GBP2 interacts with OTUD5 and promotes GPX4 ubiquitination and degradation [21]. These findings provide direct mechanistic links between immune-cell-derived signals and ferroptotic injury in distinct pulmonary parenchymal compartments.

However, the intervening links remain unresolved. It is not known whether sepsis-reprogrammed HSPCs preferentially generate CXCR4^+^ aged neutrophils, whether these neutrophils constitute the predominant NET-producing population, or whether this sequence causally contributes to parenchymal ferroptosis in SALI. Accordingly, a provisional sequence—sepsis-associated HSPC reprogramming, CXCR4^+^ aged neutrophil accumulation, NET release, METTL3-dependent disruption of GPX4 expression, and alveolar epithelial ferroptosis—can be proposed only as an inference assembled from separate studies, with each connecting step requiring direct validation. HSPC remodeling, CXCR4^+^ neutrophil accumulation, NET formation, and epithelial ferroptosis should therefore be viewed as related but not yet causally integrated processes. The proposed immunosenescence–parenchymal ferroptosis cascade represents a testable mechanistic framework rather than an established pathway. Overall, senescence-associated remodeling may impair lung recovery through complementary defects in AT2-mediated epithelial regeneration, endothelial repair, and immune homeostasis. The principal cell-specific mechanisms, senescence-associated features, functional consequences, and current evidence discussed in Section 2 and Section 3 are summarized in Table 1, providing a comparative basis for the ferroptosis–senescence crosstalk framework developed in Section 4.

## 4. Crosstalk Between Ferroptosis and Cellular Senescence in SALI

Building on the preceding analyses, ferroptosis and cellular senescence should be considered interacting, context-dependent stress programs rather than components of a fixed linear pathway in SALI [15,16,44]. Ferroptotic lipid peroxidation, mitochondrial oxidative stress, and organelle damage may promote senescence-associated responses, whereas senescence-associated alterations in iron handling, lysosomal function, antioxidant defenses, and SASP signaling may modify ferroptosis susceptibility in the same or neighboring cells [15,16,30,31]. The direction and consequences of this crosstalk are likely to depend on cell type, injury duration, and the balance between cell-autonomous adaptation and paracrine inflammatory signaling. Direct SALI evidence currently supports ferroptosis-induced senescence in pulmonary endothelial cells. In contrast, senescence-associated ferroptosis resistance, SASP-mediated paracrine ferroptosis, and the proposed temporal transition from early adaptation to later injury amplification are inferred mainly from non-pulmonary models and remain unvalidated in SALI [16,30,31,37]. Accordingly, this section examines ferroptosis-induced senescence, senescence-associated ferroptosis resistance coupled with microenvironmental amplification, and the proposed temporal evolution of this axis during SALI.

### 4.1. Ferroptosis-Induced Senescence and Shared Regulatory Nodes

Ferroptosis-induced senescence should be interpreted as a context-dependent transition rather than an inevitable consequence of ferroptotic injury [15,88,89]. Sub-lethal lipid-peroxidative and mitochondrial stress may activate DNA-damage responses and senescence-associated programs in cells that survive the initial oxidative insult [15,49,74,75,88,89]. However, whether this response remains a transient senescence-like adaptation or progresses to established cellular senescence is likely to depend on cell identity, injury intensity, redox-buffering capacity, and stress duration [15,41,74,75].

Among currently available pulmonary studies, the clearest direct evidence linking ferroptosis to senescence-associated remodeling is derived from endothelial cells [16]. In experimental sepsis-associated lung injury, ferroptotic stress promoted endothelial senescence-associated phenotypes, accompanied by impaired proliferation, increased vascular permeability, and aggravated pulmonary injury [16]. Mechanistically, the SIRT4–STAT3 pathway was implicated as a regulatory node connecting mitochondrial stress, ferroptotic susceptibility, and endothelial senescence-associated remodeling [16]. These findings provide direct in vivo evidence for an endothelial ferroptosis–senescence relationship in SALI [16].

By contrast, available AT2- and alveolar epithelial cell-focused SALI studies have primarily investigated ferroptotic injury rather than ferroptosis-induced senescence [22,65,69,70]. Recent epithelial and primary AT2-cell studies have identified ferritinophagy-associated mitochondrial iron loading and DMT1-dependent iron accumulation as regulators of ferroptotic injury [58,59]. In parallel, m6A- and lactylation-dependent pathways regulate GPX4 defense, ACSL4-mediated lipid remodeling, and SLC7A11–GSH metabolism in alveolar epithelial cells [22,65,69,70]. Experimental sepsis studies have also reported pulmonary senescence-associated or cellular-aging phenotypes at the whole-lung level [40,72]. However, none of these studies established an AT2-specific causal transition from ferroptotic stress to stable cellular senescence [22,40,58,59,65,69,70,72], as defined by persistent p16INK4a–RB activation, durable proliferative arrest, and acquisition of a stable SASP [74,75].

Mechanistic insights from non-pulmonary systems provide supporting—but indirect—evidence for shared ferroptosis–senescence regulatory nodes [15,88,89]. In renal endothelial cells and vascular smooth muscle cells, ferroptotic signaling has been experimentally associated with senescence-related phenotypes and tissue dysfunction [88,89]. These studies support the biological plausibility of ferroptosis–senescence coupling but cannot substitute for direct evidence from AT2 cells in LPS- or CLP-induced SALI models [15,16,88,89].

**Figure 2:**
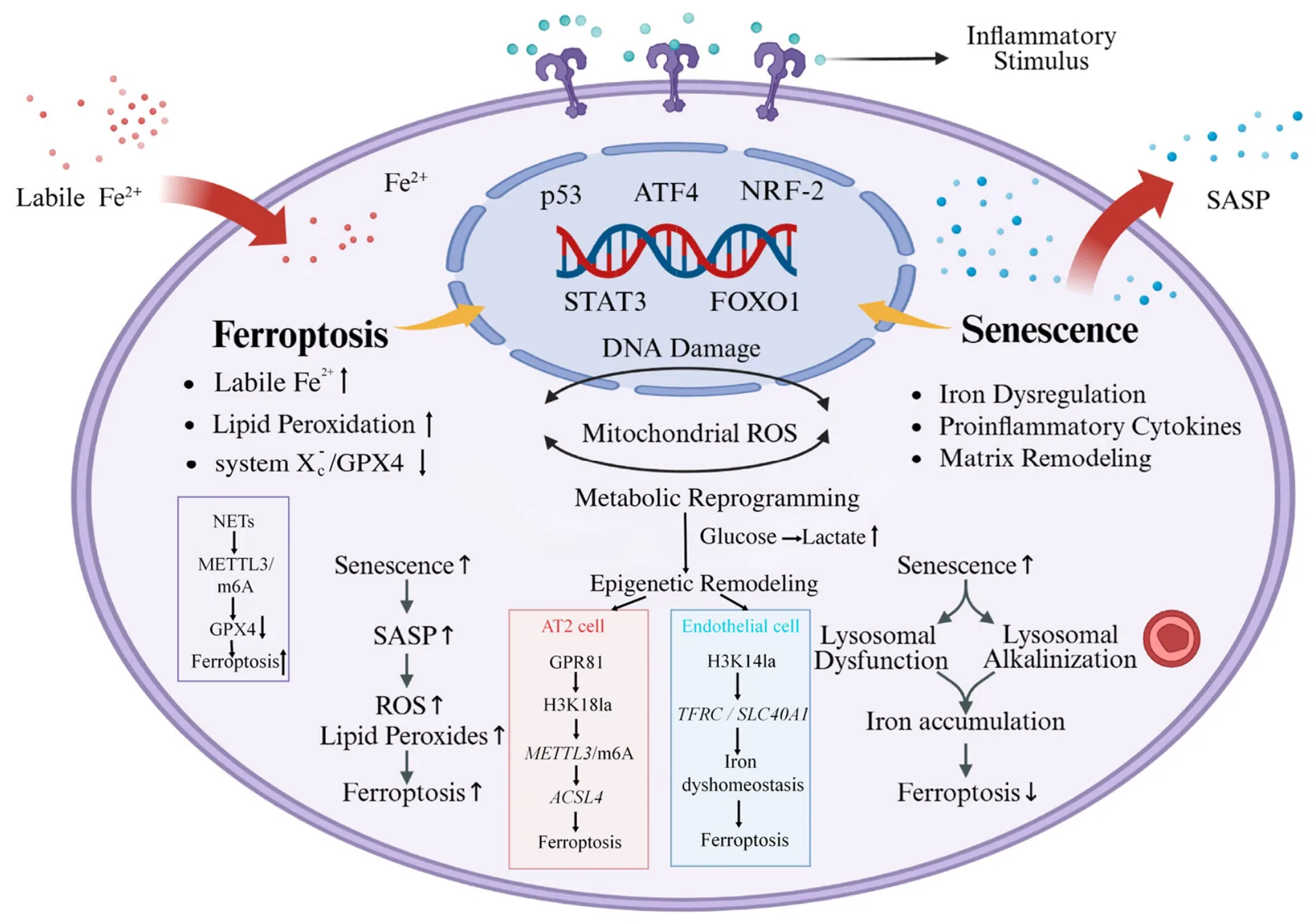
Molecular crosstalk between ferroptosis and cellular senescence in SALI. Shared stress-response nodes, including p53, ATF4, NRF2, STAT3, FOXO1, mitochondrial ROS, DNA damage, iron dyshomeostasis, metabolic reprogramming, and lysosomal dysfunction, may influence ferroptosis susceptibility and senescence-associated phenotypes. Lactate-dependent histone lactylation and m6A regulation provide cell-specific epigenetic links to ferroptosis: the GPR81–H3K18la–METTL3/m6A–ACSL4 pathway operates in alveolar epithelial cells, whereas H3K14la-dependent regulation of TFRC/SLC40A1 affects endothelial iron homeostasis. NET-associated METTL3/m6A signaling may additionally suppress GPX4 and promote epithelial ferroptosis. Gene symbols are italicized, whereas protein names and signaling nodes are shown in roman type. Arrows indicate the direction of the depicted molecular, regulatory, and phenotypic relationships between ferroptosis and cellular senescence. Created in BioRender. Chen, Q. (2026) https://BioRender.com/hxuyyj0.

Collectively, current evidence supports a hierarchical model of ferroptosis-induced senescence in SALI. Direct evidence is currently strongest for pulmonary endothelial cells, where ferroptosis inhibition attenuates senescence-associated remodeling during experimental sepsis. AT2 epithelial cells represent a biologically plausible but insufficiently validated compartment, despite extensive evidence linking epithelial ferroptosis to iron metabolism, lipid remodeling, and antioxidant failure. The proposed context-dependent molecular crosstalk between ferroptosis and cellular senescence in SALI is summarized in Figure 2. Evidence from non-pulmonary systems should therefore be interpreted as mechanistic support rather than direct validation of SALI-specific pathways.

### 4.2. Senescence-Associated Ferroptosis Resistance and Microenvironmental Amplification

Evidence for relative ferroptosis resistance in senescent cells is currently derived predominantly from non-pulmonary experimental systems. In these models, senescent cells can exhibit increased total intracellular iron while remaining relatively resistant to ferroptosis [30,31]. Impaired ferritinophagy can retain iron within ferritin, while senescence-associated lysosomal alkalinization may further sequester ferrous iron in lysosomes, reducing the redox-active iron available for cystine-deprivation-induced lipid peroxidation [30,31,90]. Restoration of lysosomal acidity can resensitize senescent cells to ferroptosis in non-pulmonary models, highlighting subcellular iron compartmentalization as a key determinant of cell fate [31,90]. However, no direct experimental study has demonstrated that senescent AT2 epithelial cells or pulmonary endothelial cells acquire an equivalent ferroptosis-resistant phenotype in LPS- or CLP-induced SALI [30,31,90]. These mechanisms should therefore be regarded as a non-pulmonary mechanistic framework rather than an established feature of septic lung injury.

Although senescent cells may acquire relative cell-autonomous resistance to ferroptosis, they can still contribute to ferroptotic damage by remodeling the surrounding microenvironment [30,31,36,37,82]. SASP factors, including cytokines, chemokines, proteases, and pro-oxidative mediators, may sustain inflammatory and oxidative signaling and thereby alter the redox-buffering capacity of neighboring epithelial and endothelial cells [35,36]. However, direct evidence that senescent-cell-derived SASP factors induce paracrine ferroptosis in pulmonary epithelial or endothelial cells during SALI is currently lacking. The finding that senescent macrophages induce ferroptosis in adjacent skeletal muscle cells provides a non-pulmonary proof of principle but does not establish an equivalent pathway within the alveolar–capillary unit [37].

Pulmonary microenvironmental amplification of ferroptosis has nevertheless been demonstrated through immune-cell-derived signals that are mechanistically distinct from the SASP [21,22]. Neutrophil extracellular traps promote METTL3–YTHDF2-dependent m6A degradation of *GPX4* mRNA and ferroptosis in alveolar epithelial cells, whereas macrophage-derived extracellular vesicles deliver GBP2 to pulmonary microvascular endothelial cells, promoting GPX4 ubiquitination and degradation, endothelial ferroptosis, and barrier dysfunction [21,22]. These pathways provide SALI-specific molecular evidence that inflammatory immune signals can amplify ferroptotic injury in the epithelial and endothelial compartments. However, neither pathway has been shown to originate from senescent cells or to be mediated by a defined SASP program.

Collectively, current evidence supports immune-mediated pulmonary microenvironmental amplification of ferroptosis, but it does not establish either cell-autonomous ferroptosis resistance in senescent AT2 or pulmonary endothelial cells or a senescent-cell-derived SASP–ferroptosis pathway in SALI. Relative ferroptosis resistance within senescent pulmonary cells and SASP-mediated ferroptotic injury in neighboring cells should therefore be regarded as two testable, cell-type-dependent hypotheses rather than established components of a continuous SALI ferroptosis–senescence pathway.

### 4.3. A Temporally Dynamic Ferroptosis–Senescence Axis in SALI

Ferroptosis–senescence crosstalk in SALI is likely to be dynamic rather than governed by a fixed unidirectional sequence. However, the available temporal evidence is fragmented and has been generated in separate experimental systems. Time-course studies of LPS-induced lung injury indicate that ferroptosis-associated changes, including decreased GPX4 and GSH-related antioxidant capacity together with increased Fe^2+^ accumulation, lipid peroxidation, and ferroptosis-related gene expression, can emerge during the first 12–24 h after model establishment [61,65,69,91,92]. By contrast, at 24 h after CLP, whole-lung p21 and the SASP-related mediator MMP3 were increased, whereas p16 was decreased and lamin B1 showed only a decreasing tendency, supporting an early p21-dominant senescence-like response rather than established p16INK4a-associated senescence [72]. Available later observations have not demonstrated a sequential transition from this early p21-associated state to persistent p16INK4a-positive senescence. Because these ferroptosis and senescence findings were obtained from different studies, models, and cellular resolutions, their occurrence during the acute phase should not be interpreted as evidence that ferroptosis necessarily precedes or causes pulmonary senescence.

With sustained inflammatory and metabolic stress, ferroptotic injury and senescence-associated remodeling may coexist, but the functions of their shared transcriptional regulators are cell- and context-dependent. In pulmonary epithelial cells, acetylated p53 can repress SLC7A11 and promote acute ferroptotic injury [28], whereas the p53–p21-mediated delay of glutathione depletion and ferroptosis has been demonstrated mainly in non-pulmonary systems and has not been validated as an early protective mechanism in SALI [29,93]. NRF2 predominantly supports acute anti-ferroptotic defense in alveolar epithelial cells through the NRF2–SLC7A11–GPX4 pathway [25,69] and in pulmonary endothelial cells through Keap1–NRF2–GPX4 signaling [94]. However, whether persistent NRF2 activation modifies established pulmonary senescence during later or non-resolving injury remains unknown. In pulmonary endothelial cells, FOXO1 nuclear activity promotes ALDH1L2 expression, fatty-acid oxidation, and mitochondrial quality control, thereby limiting ferroptosis [32]. Pro-ferroptotic FOXO1 pathways identified in non-pulmonary systems should not be extrapolated to AT2 cells or assigned to a late SALI stage without direct validation. Similarly, lactate-dependent H3K18la–METTL3–ACSL4 signaling in alveolar epithelial cells and histone lactylation-mediated ferroptosis in pulmonary endothelial cells demonstrate cell-specific metabolic regulation [50,51,65], but they do not establish a temporally ordered late-phase ferroptosis–senescence feed-forward loop.

**Figure 3:**
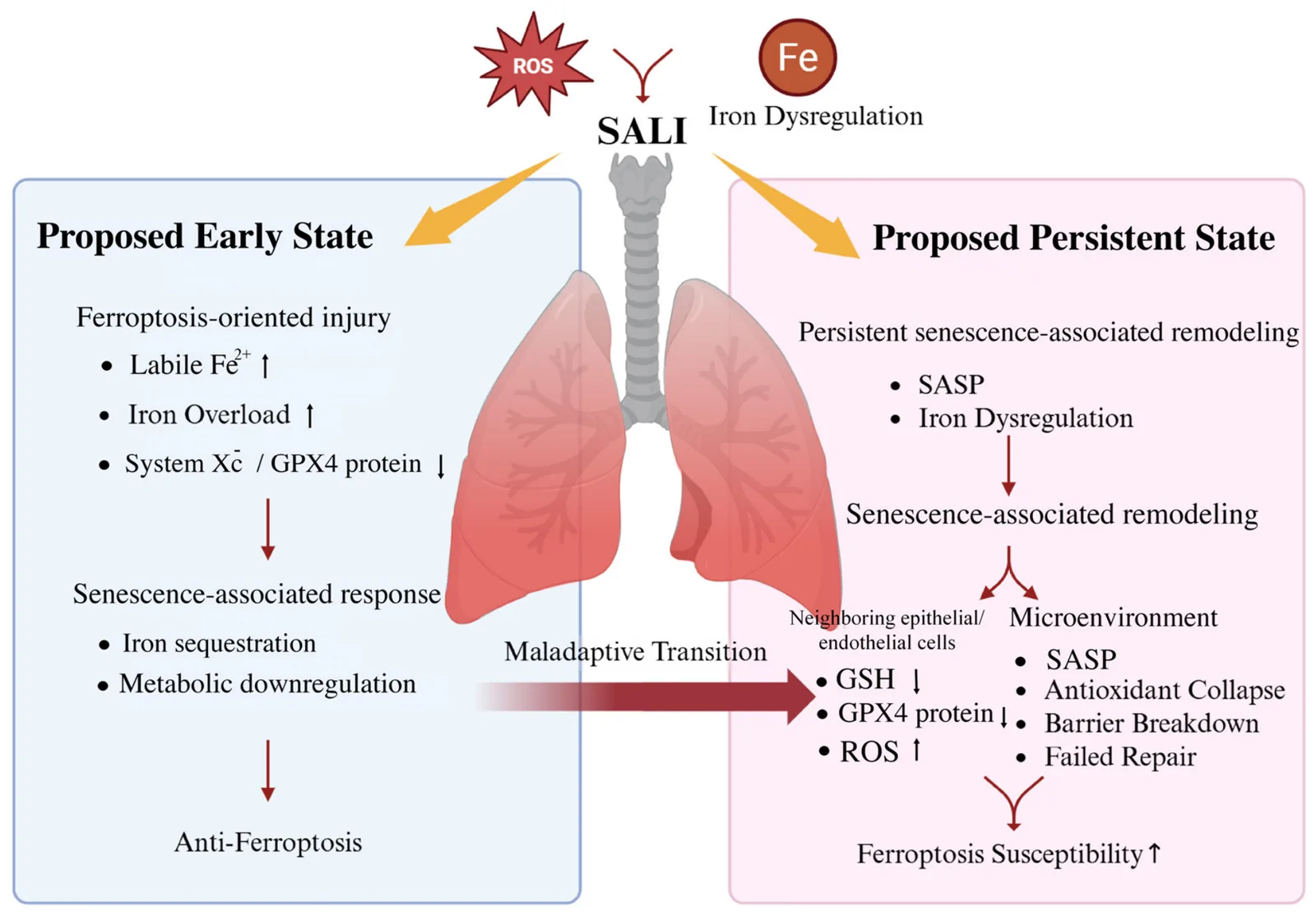
Proposed temporal model of the ferroptosis–senescence axis in SALI. Note: During early injury, ferroptosis-dominant stress may induce adaptive senescence-associated responses with iron sequestration and transient ferroptosis resistance. Persistent senescence during later stages promotes intracellular redox failure and a pro-oxidative microenvironment, thereby increasing ferroptosis susceptibility and impairing lung repair. This early-to-late transition is conceptual and has not yet been directly demonstrated in time-resolved SALI models. Arrows indicate the proposed temporal progression and directional relationships among the depicted pathological and cellular processes. Created in BioRender. Chen, Q. (2026) https://BioRender.com/sa6u8pe.

Overall, the available evidence supports the coexistence of acute ferroptotic stress and early p21-associated pulmonary senescence-like responses, but it does not establish a sequential progression toward persistent p16INK4a-positive senescence or subsequent SASP-mediated ferroptotic amplification. The proposed temporal axis should therefore be regarded as an evidence-informed but unvalidated framework. This evidence-informed temporal framework of ferroptosis–senescence crosstalk in SALI is illustrated in Figure 3. Its validation will require a single longitudinal LPS or CLP study that simultaneously measures ferroptosis markers—including GPX4, SLC7A11, ACSL4, lipid ROS, Fe^2+^, GSH, and 4-HNE—and multidimensional senescence markers—including p21, p16INK4a, γH2AX, lamin B1, proliferative capacity, and defined SASP components—within AT2 epithelial and pulmonary endothelial cells at matched early-injury, peak-injury, resolution, and persistent-injury time points. The current strength of evidence supporting individual components of the proposed ferroptosis–senescence crosstalk is summarized in Table 2. This evidence hierarchy distinguishes directly demonstrated SALI mechanisms from supportive extrapolations and currently unvalidated relationships.

## 5. Therapeutic Implications

**Figure 4:**
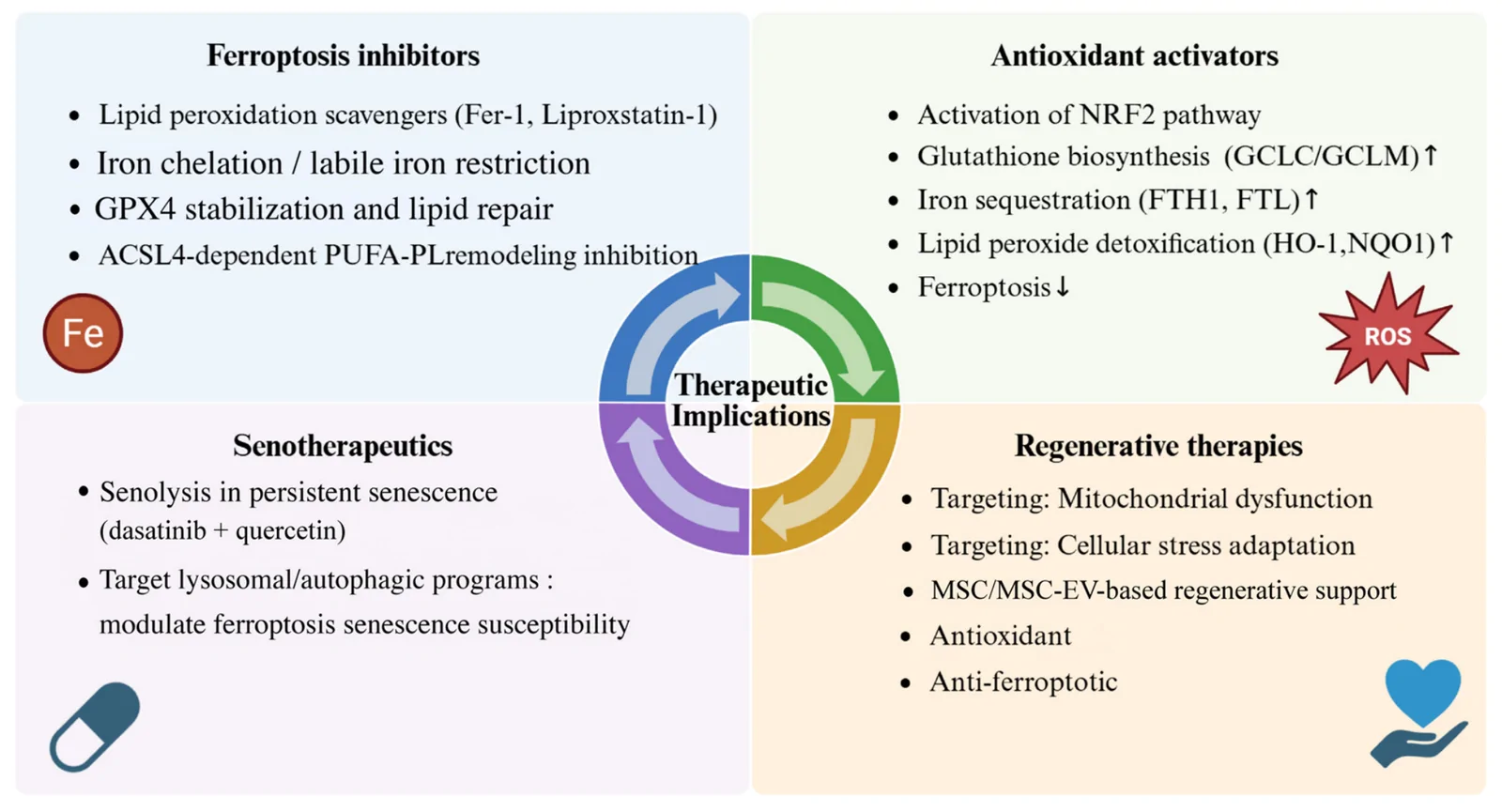
Therapeutic strategies targeting the ferroptosis–senescence axis in SALI. Note: Potential interventions include ferroptosis inhibitors, antioxidant activators, senotherapeutics, and regenerative therapies targeting mitochondrial dysfunction and stress adaptation. Effective treatment will likely require stage-specific and cell-type-specific intervention. Gene symbols are italicized where they specifically denote genes, whereas protein names and signaling pathways are shown in roman type. Circular arrows indicate complementary therapeutic strategies; ↑ and ↓ indicate increases and decreases, respectively. Created in BioRender. Chen, Q. (2026) https://BioRender.com/sztm5a0.

Building on the context-dependent ferroptosis–senescence model outlined above, therapeutic targeting in SALI should be regarded as a stage- and cell-type-informed preclinical strategy rather than an established clinical approach. Candidate interventions can be organized according to their mechanistic targets, proposed disease stage, primary target cells, strength of SALI-specific evidence, and key translational limitations. This framework distinguishes approaches supported directly in SALI from those based on partial, indirect, or extrapolated evidence, emphasizing that the proposed timing should not be interpreted as a fixed clinical treatment algorithm. Experimental studies indicate that limiting lipid peroxidation, restricting redox-active iron, preserving GPX4-dependent lipid repair, or restoring antioxidant capacity can attenuate ferroptosis-associated epithelial and endothelial injury, inflammatory activation, and alveolar–capillary leakage [60,61,62,64,95]. In parallel, modulation of persistent senescence-associated inflammation or enhancement of reparative programs may become relevant when acute injury evolves into defective resolution. Accordingly, the therapeutic framework shown in Figure 4 comprises four complementary approaches: direct ferroptosis inhibition, activation of endogenous antioxidant defenses, senotherapeutic modulation, and regenerative interventions targeting mitochondrial dysfunction and stress adaptation. The central challenge will be to match each intervention to the dominant injury program, cellular target, disease stage, and level of supporting evidence.

Clinical translation will require biomarker-guided patient enrichment. Existing human markers mainly define inflammatory and alveolar–capillary injury phenotypes rather than ferroptosis- or senescence-dominant SALI. Plasma classifiers incorporating IL-6, IL-8, soluble TNF receptor-1, protein C, bicarbonate, and vasopressor use distinguish hyperinflammatory and hypoinflammatory phenotypes, whereas sRAGE, SP-D, and CC16 reflect epithelial injury, and angiopoietin-2 and von Willebrand factor reflect endothelial involvement [11,96,97,98,99]. BALF may provide complementary compartment-specific information [100]. Longitudinal studies indicate relative phenotype stability during the first three days and associations between serial injury-marker trajectories and mortality, but no validated panel currently identifies ferroptosis- or senescence-dominant SA-ARDS [99,101]. Candidate exploratory markers include 4-HNE, MDA, iron indices, GSH/GPX4-related readouts, p21, and SASP proteins; their specificity, temporal behavior, reproducibility, and relationship to pulmonary cell states require prospective validation.

Among early ferroptosis-directed approaches, reinforcement of endogenous antioxidant and lipid-repair systems represents a complementary strategy to direct radical-trapping agents. NRF2 coordinates cystine utilization, glutathione biosynthesis, iron buffering, and antioxidant gene expression, thereby raising the threshold for phospholipid-peroxide accumulation [25,26]. In experimental sepsis-induced lung injury, the RNA-binding protein AUF1 counteracts ferroptosis through differential regulation of NRF2 and ATF3 transcript stability; loss of this pathway weakens endogenous redox protection [25]. FSP1 provides an additional GPX4-independent CoQ-based defense against lipid peroxidation [102,103]. Recent evidence further implicates STING–FSP1 signaling in septic endothelial ferroptosis and vascular leakage [104]. Thus, pharmacological enhancement of NRF2 signaling or preservation of FSP1 activity may support endothelial redox resilience and barrier function during early injury. However, because these pathways also influence inflammatory and host-defense responses, their timing, cell specificity, and safety require validation in clinically relevant SA-ARDS models. Translation of ferroptosis-directed therapy is limited by the absence of clinically validated ferroptosis-selective dosing strategies, uncertain pulmonary pharmacokinetics, and inadequate cell-specific delivery. Systemic iron chelation may disturb physiological iron availability, whereas broad modulation of NRF2, STING, or lipid-peroxidation pathways may alter antimicrobial defense, inflammatory signaling, and tissue repair. These approaches therefore require pharmacodynamic biomarkers and early-phase dose-ranging studies rather than direct extrapolation from rodent efficacy.

Beyond cell-intrinsic anti-ferroptotic programs, interruption of pathogenic intercellular signaling represents an additional, but still exploratory, therapeutic direction in SALI. In experimental SALI, macrophage-derived extracellular vesicles deliver guanylate-binding protein 2 (GBP2) to pulmonary microvascular endothelial cells [21]. GBP2 interacts with OTUD5 and promotes GPX4 ubiquitination and degradation, thereby enhancing endothelial ferroptosis and vascular barrier dysfunction [21]. This pathway identifies several potential intervention points, including selective blockade of pathogenic vesicle release or uptake, neutralization of EV-associated GBP2, and preservation of GPX4-dependent lipid repair in recipient endothelial cells. However, extracellular vesicles can also convey reparative signals during sepsis, and the EV–GBP2 axis has not been therapeutically validated in human SA-ARDS [105]. EV-directed intervention should therefore be regarded as a cargo-specific, cell-targeted preclinical strategy rather than a nonspecific approach to suppress extracellular vesicle signaling. Clinical implementation would additionally require standardized assays capable of distinguishing pathogenic EV subpopulations and quantifying disease-relevant cargo such as GBP2. Nonspecific suppression of EV release or uptake would be difficult to justify because it could simultaneously eliminate reparative intercellular signals.

When acute lipid-peroxidative injury evolves into persistent senescence-associated inflammation and defective repair, senotherapeutic approaches may become relevant as adjunctive strategies. Senotherapeutics include senolytics, which selectively eliminate senescent cells, and senomorphics, which suppress deleterious senescence-associated secretory phenotypes without cell removal [43,106]. Accordingly, senolytic intervention should be considered only when persistent, maladaptive cellular senescence is supported by longitudinal and multidimensional evidence, rather than by isolated p21 elevation or other acute stress-associated markers. In a CLP model, dasatinib plus quercetin reduced mortality and senescence-associated changes in the liver; however, this intervention was administered before sepsis induction and does not establish therapeutic efficacy in established SALI [107]. Clinical translation remains limited. No completed therapeutic trial has yet demonstrated efficacy of a ferroptosis-specific intervention in sepsis or ARDS, and current ferroptosis-related clinical research is primarily biomarker-oriented rather than therapeutic. The phase II STOP-Sepsis trial (NCT05758246) is evaluating the senolytic fisetin in older adults with sepsis, but it is not specific to SALI or ARDS and has not yet reported efficacy results [108]. ARDS trials of MSC-based therapies should therefore be regarded as indirect immunomodulatory or regenerative translation rather than direct clinical validation of the ferroptosis–senescence axis [108]. Modulation of lysosomal and autophagic programs offers an additional experimental approach [31,90]. In non-pulmonary senescence models, lysosomal alkalinization contributes to resistance to cystine-deprivation-induced ferroptosis, whereas restoration of lysosomal acidity can resensitize senescent cells to ferroptotic death [31,90]. Whether this ferroptosis-based senolytic strategy can selectively eliminate pathogenic senescent pulmonary cells without worsening epithelial or endothelial injury remains unknown [31,90]. Thus, senotherapeutic intervention in SALI should be considered stage-dependent and highly exploratory, with careful attention to cellular targets, host-defense competence, and repair requirements. Major translational barriers include the absence of a validated biomarker for identifying persistent pathogenic senescent cells in the lung, uncertainty regarding the therapeutic window, and the risk of eliminating transiently arrested epithelial or endothelial cells that retain reparative potential. Systemic toxicities and interactions with antimicrobial immunity must also be evaluated separately for each senolytic or senomorphic agent.

In contrast to pathogenic extracellular vesicle signaling, mesenchymal stromal/stem cell (MSC)-based and MSC-derived extracellular vesicle (EV) therapies may offer a repair-oriented strategy to modulate ferroptosis in SALI. In preclinical sepsis models, MSC administration reduced neutrophil extracellular trap formation and pulmonary ferroptosis, whereas adipose-derived MSC EVs suppressed macrophage ferroptosis through SIRT1–NRF2 signaling and protected pulmonary microvascular endothelial cells through the miR-125b-5p–Keap1–NRF2–GPX4 pathway [94,109,110].

Importantly, MSC therapy has entered early-phase ARDS trials, but efficacy in SA-ARDS remains unproven. The START and REALIST studies supported the feasibility of intravenous allogeneic MSC administration, although neither established a survival benefit; START also revealed substantial variability in post-thaw cell viability [111,112]. BALF biomarker reductions after MSC treatment suggested local biological activity without demonstrating ferroptosis-specific target engagement [113]. Major barriers include uncertain delivery route, dose, biodistribution, and dosing frequency; donor and product heterogeneity; culture, cryopreservation, viability, and release criteria; and possible alloimmunization [111,114]. MSC-derived EVs additionally require scalable GMP production, standardized purification and dosing, cargo stability, storage, and validated potency assays. A pilot trial in COVID-19-associated ARDS did not establish clear clinical benefit and cannot be directly extrapolated to bacterial SA-ARDS [115]. Thus, MSC therapy is clinically initiated but investigational, whereas ferroptosis-targeted MSC-EV therapy remains predominantly preclinical. The principal candidate interventions discussed above, together with their molecular targets, proposed therapeutic stages, SALI-specific evidence, and current level of experimental or clinical development, are summarized in Table 3.

Collectively, therapeutic targeting of ferroptosis–senescence crosstalk in SALI should be guided by disease stage, cellular target, and the dominant injury program rather than by a fixed treatment sequence. During acute lipid-peroxidative injury, ferroptosis inhibitors and reinforcement of endogenous antioxidant defenses may help preserve epithelial and endothelial barrier function. When persistent senescence-associated inflammation and defective repair become prominent, senomorphic, senolytic, or regenerative approaches may become relevant. However, clinical translation is constrained by the absence of validated ferroptosis–senescence companion diagnostics, uncertain therapeutic windows, inadequate pulmonary and cell-specific delivery, poorly defined pharmacokinetics, and potential interference with antimicrobial immunity or physiological repair. A provisional stratification approach could combine lipid-peroxidation products, iron indices, and GSH–GPX4 status as a ferroptosis-oriented profile, whereas persistent cell-associated p21/p16 expression and SASP mediators could support a senescence-oriented profile, interpreted alongside epithelial and endothelial injury markers in serial plasma or BALF samples. These are exploratory composite signatures rather than validated phase-defining tests, and no current threshold reliably classifies individual patients as “ferroptosis-dominant” or “senescence-dominant” [25,26,35,49,72,96,116,117].

Future clinical development should combine biomarker-based enrichment with serial plasma and, where feasible, bronchoalveolar lavage sampling. Mechanistic endpoints should be linked to established clinical outcomes, including ventilator-free days, organ-support requirements, mortality, and long-term pulmonary function. For cell- and EV-based therapies, donor selection, manufacturing consistency, potency assays, dosing, administration route, biodistribution, and alloimmune monitoring should be prespecified. These measures will be necessary to determine whether molecularly selected SA-ARDS subgroups derive benefit from mechanism-guided treatment rather than exposing an unselected heterogeneous population to biologically active but insufficiently targeted interventions.

## 6. Conclusions

Ferroptosis and cellular senescence should be considered interacting, context-dependent stress programs in SALI rather than isolated processes or components of a fixed linear pathway. Shared disturbances, including oxidative stress, mitochondrial dysfunction, iron dyshomeostasis, and inflammation-associated metabolic reprogramming, can converge on pulmonary epithelial and endothelial cells, thereby influencing cell survival, alveolar–capillary barrier integrity, inflammatory signaling, and tissue repair [15,17,18,20]. Current experimental evidence supports a functional link between ferroptotic stress and endothelial senescence in septic lung injury, whereas the reverse relationship, including senescence-associated ferroptosis resistance and paracrine amplification, is supported mainly by non-pulmonary models [16,30,31,37]. We therefore propose a temporally and spatially heterogeneous ferroptosis–senescence framework in which cell-autonomous adaptation can coexist with microenvironmental injury amplification. This framework offers a useful lens for interpreting SALI pathogenesis but requires time-resolved and cell-specific validation.

Several limitations preclude firm conclusions regarding ferroptosis–senescence crosstalk in SALI. First, existing mechanistic evidence is derived predominantly from preclinical studies conducted at a limited number of time points, and the proposed transition from early cell-autonomous adaptation to later tissue-level injury amplification has not been directly tested in time-resolved SALI models [72,118]. Although senescence-associated responses have been detected in septic lungs and ferroptotic stress has been linked to endothelial senescence, the temporal order, persistence, and causal coupling of these events remain unresolved [16,72]. Second, epithelial, endothelial, and immune compartments are likely to differ in ferroptosis sensitivity and senescence-associated phenotypes, yet their cell-specific interactions have not been systematically delineated [16,21,22,118]. Third, longitudinal human SA-ARDS datasets integrating molecular phenotypes with clinical trajectories and treatment responses remain scarce [96,116,117]. These gaps currently preclude the definition of universal biomarkers or fixed stage-specific intervention algorithms.

Future research should prioritize four coordinated and experimentally testable validation strategies. First, tamoxifen-inducible *Sftpc*-CreER- and *Cdh5*-CreERT2-based lineage-tracing systems should be applied in time-resolved LPS and CLP models to define the fate of AT2 epithelial and pulmonary endothelial cells. Lineage-resolved assessment of ferroptosis, multidimensional senescence markers, and cell-specific manipulation of *Gpx4*, *Slc7a11*, or *Cdkn1a* would help establish the causal direction of ferroptosis–senescence coupling rather than relying on marker colocalization alone [16,25,28,69,72,80,81,118,119,120,121].

Second, sequential treatment studies should compare early ferroptosis inhibition with delayed senomorphic or senolytic intervention, simultaneous treatment, and stage-adapted combination therapy. These studies should evaluate lung injury, survival, microbial clearance, systemic toxicity, and epithelial–endothelial regeneration, with proposed treatment windows regarded as testable intervals rather than established schedules [60,61,62,94,107,108,109,110]. Third, prospective multicenter cohorts should combine serial plasma and clinically indicated bronchoalveolar lavage sampling with composite inflammatory, epithelial–endothelial injury, ferroptosis, and senescence-associated biomarkers, and relate their trajectories to organ support, mortality, and long-term pulmonary outcomes [11,96,97,98,99,100,101,116,117]. Finally, primary human AT2 organoids and multicellular alveolar–capillary models incorporating endothelial and immune cells should be used to validate cell-specific and paracrine mechanisms and to test sequential interventions in a human-relevant pulmonary microenvironment before clinical translation [25,28,32,65,69,120,122,123,124].

## Figures and Tables

**Table 1:** Cell-specific summary of ferroptosis and senescence in sepsis-associated acute lung injury.

Cell Type	Principal Ferroptosis-Related Mechanisms	Senescence-Associated Features/Biomarkers	Functional Consequences	Interpretation in SALI	Representative Evidence
AT2 epithelial cells	NCOA4-dependent ferritinophagy and mitochondrial iron loading; DMT1-dependent iron accumulation; NET–METTL3/m6A–GPX4 signaling; H3K18la–METTL3–ACSL4 and LPCAT-related lipid remodeling; SLC7A11–GSH–GPX4 impairment	p21, p16INK4a, γH2AX, lamin B1 loss, SA-β-gal, reduced Ki67/EdU, SASP; persistence should be demonstrated longitudinally	Ferroptotic epithelial loss, impaired organoid formation and AT2-to-AT1 differentiation, defective alveolar repair	Direct evidence for AT2 ferroptosis is substantial, whereas direct cell-resolved evidence for established AT2 senescence and ferroptosis-induced senescence remains limited	[22,40,58,59,65,69,70,72]
Pulmonary endothelial cells	EV–GBP2–OTUD5–GPX4 signaling; iron-transport dysregulation; DPEP1–FOXO1–ALDH1L2; FUNDC1–GPX4-associated mitochondrial quality control	p21/p16INK4a, γH2AX, lamin B1 loss, SA-β-gal, reduced proliferation, endothelial SASP	Increased permeability, vascular leakage, impaired endothelial regeneration and barrier repair	Strongest direct SALI evidence for ferroptosis-associated senescence remodeling is currently derived from endothelial cells	[16,21,32,50,51,71]
Alveolar macrophages	Macrophage-derived EVs can amplify endothelial ferroptosis through GBP2–OTUD5–GPX4 signaling	p21/p16INK4a, γH2AX, SA-β-gal and SASP may be informative, but inflammatory activation alone is insufficient to define senescence	Cytokine release, phagocytosis/efferocytosis changes and paracrine injury	Macrophage activation may mimic senescence-associated phenotypes; multidimensional criteria are required	[21,35,36]
Neutrophils	NETs promote METTL3-dependent m6A regulation of GPX4 and epithelial ferroptosis	CXCR4 elevation, prolonged survival and altered trafficking indicate an aged-neutrophil phenotype rather than canonical senescence	Tissue retention, delayed apoptosis and NET formation	Direct NET-to-epithelial ferroptosis is supported, but CXCR4^+^ aged neutrophils should not be classified as canonical senescent cells	[22,83]
HSPCs	No direct pulmonary ferroptosis pathway has been established	Persistent functional and myeloid reprogramming rather than canonical pulmonary senescence markers	Altered self-renewal, mobilization, lineage output and myeloid bias	Represents systemic immune remodeling; a causal HSPC–neutrophil–ferroptosis cascade remains unproven	[84,85,86,87]

Note: No single biomarker is sufficient to establish cellular senescence. Established senescence requires durable growth arrest beyond the initiating insult together with concordant multidimensional evidence, such as persistent p16INK4a–RB activation, DNA-damage signaling, lamin B1 loss, SA-β-gal activity, and a defined SASP. CXCR4^+^ neutrophil aging and HSPC reprogramming should not be equated with canonical cellular senescence. AT2, alveolar type II; HSPC, hematopoietic stem and progenitor cell; NET, neutrophil extracellular trap; SA-β-gal, senescence-associated β-galactosidase; SASP, senescence-associated secretory phenotype.

**Table 2:** Evidence hierarchy for the proposed ferroptosis–senescence crosstalk in SALI.

Proposed Relationship	Principal Cell Compartment	Available Evidence	Evidence Status in SALI	Interpretation
Ferroptosis → senescence-associated remodeling	Pulmonary endothelial cells	Experimental SALI evidence linking ferroptotic stress with senescence-associated phenotypes, impaired proliferation and vascular dysfunction	Direct SALI evidence	Currently the strongest cell-specific evidence supporting ferroptosis–senescence coupling
Ferroptosis → established senescence	AT2 epithelial cells	Extensive SALI evidence for epithelial ferroptosis and separate evidence for pulmonary senescence-associated phenotypes	Not directly demonstrated	Biologically plausible, but a causal AT2 ferroptosis-to-senescence transition remains unproven
Senescent-cell ferroptosis resistance	Primarily non-pulmonary cells	Ferritin retention, impaired ferritinophagy and lysosomal iron sequestration reported in non-pulmonary models	No direct SALI evidence	Should be regarded as an extrapolated mechanistic hypothesis
SASP → paracrine ferroptosis	Neighboring parenchymal cells	Proof-of-principle evidence predominantly from non-pulmonary systems	No direct SALI evidence	A testable pulmonary hypothesis rather than an established SALI mechanism
Immune microenvironment → parenchymal ferroptosis	Neutrophil → epithelium; macrophage → endothelium	NET–METTL3/m6A–GPX4 and EV–GBP2–OTUD5–GPX4 pathways	Direct SALI evidence	Demonstrates pulmonary microenvironmental amplification, but not a senescent-cell-derived SASP pathway

Note: Evidence categories reflect the degree of SALI-specific experimental validation rather than general biological plausibility. “Direct SALI evidence” indicates that the proposed relationship has been experimentally demonstrated in sepsis-associated lung injury models; “indirect/supportive evidence” refers to findings from related pulmonary or non-pulmonary systems that support mechanistic plausibility but do not establish the pathway in SALI; and “conceptual/unvalidated” indicates a relationship inferred from separate studies that still requires direct, cell-specific, and time-resolved validation. SALI, sepsis-associated acute lung injury; AT2, alveolar type II; SASP, senescence-associated secretory phenotype; NET, neutrophil extracellular trap; EV, extracellular vesicle.

**Table 3:** Candidate stage- and cell-informed therapeutic strategies for targeting the ferroptosis–senescence axis in SALI.

Candidate Strategy	Representative Target/Agent	Primary Compartment	Mechanistic Objective	Proposed Stage	Evidence in SALI	Evidence Stage
Labile iron restriction	Iron chelation; iron sequestration	Epithelial/endothelial	Reduce redox-active iron and Fenton-driven lipid peroxidation	Acute injury	Preclinical SALI support	Cell culture; animal models
Lipid radical trapping	Ferrostatin-1; liproxstatin-1	Epithelial/endothelial	Interrupt lipid-peroxide chain reactions and preserve membrane integrity	Acute injury	Direct experimental support	Cell culture; animal models
System Xc^−^–GSH–GPX4 restoration	Cystine/GSH support; GPX4-centered defense	Mainly epithelial/endothelial	Restore lipid-peroxide detoxification	Acute to intermediate	Direct mechanistic support	Cell culture; animal models
NRF2-centered redox enhancement	NRF2-related antioxidant signaling	Epithelial/endothelial	Enhance GSH synthesis, iron sequestration and peroxide detoxification	Acute injury	Preclinical SALI support	Cell culture; animal models
FSP1-centered ferroptosis defense	FSP1-related pathway modulation	Mainly endothelial	Complement GPX4-independent lipid antioxidant defense	Acute injury	Emerging SALI evidence	Cell culture; animal models
Immune-to-parenchymal blockade	NET inhibition; METTL3/m6A targeting; EV/GBP2 interception	Neutrophil–epithelial/macrophage–endothelial	Interrupt extrinsic GPX4-destabilizing signals	Acute to intermediate	Direct mechanistic SALI evidence	Cell culture; animal models
Metabolic/epigenetic modulation	Lactylation, m6A, ACSL4/LPCAT-related pathways	Epithelial/endothelial	Reduce PUFA-PL remodeling and restore redox metabolism	Acute to intermediate	Emerging experimental evidence	Cell culture; animal models
Senomorphic intervention	SASP- and stress-signaling modulation	Persistent senescent compartments	Suppress maladaptive secretory remodeling without cell elimination	Persistent/non-resolving injury	Limited SALI-specific evidence	Cell culture; animal models
Senolysis	Fisetin; dasatinib + quercetin	Persistently senescent cells	Selectively eliminate persistent maladaptive senescent cells	Delayed/persistent injury	SALI-specific efficacy unproven	Animal models; ongoing early clinical investigation
Lysosomal–mitochondrial modulation	Ferritinophagy, lysosomal acidity, mitochondrial quality control	Cell-dependent	Normalize iron compartmentalization and mitochondrial redox homeostasis	Context-dependent	Emerging/indirect	Cell culture; animal models
MSC/MSC-EV regenerative support	MSCs; MSC-derived EVs	Alveolar–capillary unit	Promote epithelial/endothelial recovery and immunomodulation	Repair phase	Ferroptosis–senescence-specific action remains preclinical	Cell culture; animal models; MSC clinical studies

Note: The proposed therapeutic stages are conceptual and should be regarded as testable intervention windows rather than established clinical schedules. “Evidence stage” indicates the highest level of development currently reached by the representative strategy. Clinical studies not specifically designed to target the ferroptosis–senescence axis are considered indirect translational evidence. Most ferroptosis-directed and senescence-directed interventions remain preclinical, and no biomarker-guided treatment algorithm has yet been validated in SALI or SA-ARDS. SALI, sepsis-associated acute lung injury; SA-ARDS, sepsis-associated acute respiratory distress syndrome; GSH, glutathione; GPX4, glutathione peroxidase 4; FSP1, ferroptosis suppressor protein 1; NET, neutrophil extracellular trap; EV, extracellular vesicle; MSC, mesenchymal stromal/stem cell; MSC-EV, mesenchymal stromal/stem cell-derived extracellular vesicle.

## Data Availability

No new data were created or analyzed in this study. Data sharing is not applicable to this article.

## Abbreviations

The following abbreviations are used in this manuscript:

ALI	Acute lung injury
AMPK	AMP-activated protein kinase
ARDS	Acute respiratory distress syndrome
AT2	Alveolar type II epithelial cell
ATF3	Activating transcription factor 3
ATF4	Activating transcription factor 4
AUF1	AU-rich element RNA-binding protein 1
CLP	Cecal ligation and puncture
CXCR4	C-X-C chemokine receptor type 4
DAMPs	Damage-associated molecular patterns
DPEP1	Dipeptidase 1
ECM	Extracellular matrix
FSP1	Ferroptosis suppressor protein 1
FOXO1	Forkhead box O1
FoxM1	Forkhead box protein M1
GBP2	Guanylate-binding protein 2
GPX4	Glutathione peroxidase 4
GSH	Glutathione
HMOX1	Heme oxygenase 1
I/R	Ischemia/reperfusion
JAM-C	Junctional adhesion molecule C
LPS	Lipopolysaccharide
METTL3	Methyltransferase-like 3
m6A	N6-methyladenosine
NETs	Neutrophil extracellular traps
NRF2	Nuclear factor erythroid 2
PAMPs	Pathogen-associated molecular patterns
PUFA-PLs	Polyunsaturated fatty acid-containing phospholipids
ROS	Reactive oxygen species
SALI	Sepsis-associated acute lung injury
SASP	Senescence-associated secretory phenotype
SIRT1	Sirtuin 1
SLC7A11	Solute carrier family 7 member 11
STAT3	Signal transducer and activator of transcription 3
STING	Stimulator of interferon genes
SA-ARDS	Sepsis-associated acute respiratory distress syndrome
NF-κB	Nuclear factor kappa B
HSPCs	Hematopoietic stem and progenitor cells
BALF	Bronchoalveolar lavage fluid
EV	Extracellular vesicle
MSC	Mesenchymal stromal/stem cell
ACSL4	Acyl-CoA synthetase long-chain family member 4
DMT1	Divalent metal transporter 1
NCOA4	Nuclear receptor coactivator 4
NINJ1	Ninjurin 1
OTUD5	OTU deubiquitinase 5
ALDH1L2	Aldehyde dehydrogenase 1 family member L2
FUNDC1	FUN14 domain-containing protein 1
4-HNE	4-Hydroxynonenal
MDA	Malondialdehyde

## References

[1] Singer M., Deutschman C.S., Seymour C.W., Shankar-Hari M., Annane D., Bauer M., Bellomo R., Bernard G.R., Chiche J.-D., Coopersmith C.M. (2016). The third international consensus definitions for sepsis and septic shock (sepsis-3). JAMA.

[2] GBD 2021 Global Sepsis Collaborators (2025). Global, regional, and national sepsis incidence and mortality, 1990–2021: A systematic analysis. Lancet Glob. Health.

[3] Matthay M.A., Arabi Y., Arroliga A.C., Bernard G., Bersten A.D., Brochard L.J., Calfee C.S., Combes A., Daniel B.M., Ferguson N.D. (2024). A New Global Definition of Acute Respiratory Distress Syndrome. Am. J. Respir. Crit. Care Med..

[4] Qiao X., Yin J., Zheng Z., Li L., Feng X. (2024). Endothelial cell dynamics in sepsis-induced acute lung injury and acute respiratory distress syndrome: Pathogenesis and therapeutic implications. Cell Commun. Signal..

[5] Xu X., Zhang Q., Lv Z., Cheng C., Zha J., Shu H., Xiao H., Pan S. (2025). Unraveling the deadly dance: Endothelial cells and neutrophils in sepsis-induced acute lung injury/acute respiratory distress syndrome. Front. Cell Dev. Biol..

[6] Su Y., Lucas R., Fulton D.J., Verin A.D. (2024). Mechanisms of pulmonary endothelial barrier dysfunction in acute lung injury and acute respiratory distress syndrome. Chin. Med. J. Pulm. Crit. Care Med..

[7] Wick K.D., Ware L.B., Matthay M.A. (2024). Acute respiratory distress syndrome. BMJ.

[8] Meyer N.J., Gattinoni L., Calfee C.S. (2021). Acute respiratory distress syndrome. Lancet.

[9] Bellani G., Laffey J.G., Pham T., Fan E., Brochard L., Esteban A., Gattinoni L., Van Haren F., Larsson A., McAuley D.F. (2016). Epidemiology, patterns of care, and mortality for patients with acute respiratory distress syndrome in intensive care units in 50 countries. JAMA.

[10] Prescott H.C.M., Antonelli M., Alhazzani W.M., Møller M.H., Alshamsi F.M., Azevedo L.C.P., Belley-Cote E., De Waele J., Derde L., Dionne J.C.M. (2026). Surviving Sepsis Campaign: International Guidelines for Management of Sepsis and Septic Shock 2026. Crit. Care Med..

[11] Calfee C.S., Delucchi K., Parsons P.E., Thompson B.T., Ware L.B., Matthay M.A. (2014). Subphenotypes in acute respiratory distress syndrome: Latent class analysis of data from two randomised controlled trials. Lancet Respir. Med..

[12] Wang L., Zhang W., Feng X. (2025). Ferroptosis in sepsis induced acute lung injury/acute respiratory distress syndrome (ALI/ARDS): A potential therapeutic strategy. Front. Immunol..

[13] Bao Y., Yang X., Zhao P., Du X. (2025). Research advances on the role of programmed endothelial cell death in sepsis. Cell Death Discov..

[14] Shen Y., He Y., Pan Y., Liu L., Liu Y., Jia J. (2024). Role and mechanisms of autophagy, ferroptosis, and pyroptosis in sepsis-induced acute lung injury. Front. Pharmacol..

[15] Coradduzza D., Congiargiu A., Chen Z., Zinellu A., Carru C., Medici S. (2023). Ferroptosis and senescence: A systematic review. Int. J. Mol. Sci..

[16] Yao Y., Wang S., Shi W., Yang L., Tian Y., Wang D., Xia D. (2025). Ferroptosis promotes sepsis-related lung injury via endothelial cell senescence. Cell Signal.

[17] Stockwell B.R., Angeli J.P.F., Bayir H., Bush A.I., Conrad M., Dixon S.J., Fulda S., Gascón S., Hatzios S.K., Kagan V.E. (2017). Ferroptosis: A regulated cell death nexus linking metabolism, redox biology, and disease. Cell.

[18] Tang D., Chen X., Kang R., Kroemer G. (2021). Ferroptosis: Molecular mechanisms and health implications. Cell Res..

[19] Dixon S.J., Lemberg K.M., Lamprecht M.R., Skouta R., Zaitsev E.M., Gleason C.E., Patel D.N., Bauer A.J., Cantley A.M., Yang W.S. (2012). Ferroptosis: An Iron-Dependent Form of Nonapoptotic Cell Death. Cell.

[20] Ru Q., Li Y., Chen L., Wu Y., Min J., Wang F. (2024). Iron homeostasis and ferroptosis in human diseases: Mechanisms and therapeutic prospects. Signal Transduct. Target. Ther..

[21] Li Z., Bu Y., Wang C., Yu Y., Han L., Liu C., Chen G., Li C., Zhang Y., Cao H. (2025). Extracellular vesicle-packaged GBP2 from macrophages aggravates sepsis-induced acute lung injury by promoting ferroptosis in pulmonary vascular endothelial cells. Redox Biol..

[22] Zhang H., Liu J., Zhou Y., Qu M., Wang Y., Guo K., Shen R., Sun Z., Cata J.P., Yang S. (2022). Neutrophil extracellular traps mediate m6A modification and regulates sepsis-associated acute lung injury by activating ferroptosis in alveolar epithelial cells. Int. J. Biol. Sci..

[23] Yu T., Sun S. (2023). Role and mechanism of ferroptosis in acute lung injury. Cell Cycle.

[24] Wang X., Wei T., Luo J., Lang K., Song Y., Ning X., Chao Y., Gu Z., Wang L., Chen C. (2024). Iron Overload–Dependent Ferroptosis Aggravates LPS-Induced Acute Lung Injury by Impairing Mitochondrial Function. Inflammation.

[25] Wang Y., Chen D., Xie H., Jia M., Sun X., Peng F., Guo F., Tang D. (2022). AUF1 protects against ferroptosis to alleviate sepsis-induced acute lung injury by regulating NRF2 and ATF3. Cell. Mol. Life Sci..

[26] Hou H., Qin X., Li G., Cui Z., Zhang J., Dong B., Wang Z., Zhao H. (2024). NRF2-mediated redox balance alleviates LPS-induced vascular endothelial cell inflammation by inhibiting endothelial cell ferroptosis. Sci. Rep..

[27] Gao N., Tang A.-L., Liu X.-Y., Chen J., Zhang G.-Q. (2023). p53-Dependent ferroptosis pathways in sepsis. Int. Immunopharmacol..

[28] Yang Y., Ma Y., Li Q., Ling Y., Zhou Y., Chu K., Xue L., Tao S. (2022). STAT6 inhibits ferroptosis and alleviates acute lung injury via regulating P53/SLC7A11 pathway. Cell Death Dis..

[29] Xu R., Wang W., Zhang W. (2023). Ferroptosis and the bidirectional regulatory factor p53. Cell Death Discov..

[30] Masaldan S., Clatworthy S.A., Gamell C., Meggyesy P.M., Rigopoulos A.-T., Haupt S., Haupt Y., Denoyer D., Adlard P.A., Bush A.I. (2018). Iron accumulation in senescent cells is coupled with impaired ferritinophagy and inhibition of ferroptosis. Redox Biol..

[31] Loo T.M., Zhou X., Tanaka Y., Sugawara S., Yamauchi S., Kawasaki H., Matsuoka Y., Sugiura Y., Sakuma S., Yamanishi Y. (2025). Senescence-associated lysosomal dysfunction impairs cystine deprivation-induced lipid peroxidation and ferroptosis. Nat. Commun..

[32] Yao X., Wen Z., Liao J., Meng Y., Lu P., Li X., Shen Z., Wang A., Wu M., Li X. (2026). DPEP1 mediates regulation of mitochondrial quality control via FOXO1/ALDH1L2 axis to attenuate ferroptosis in pulmonary endothelial cells to alleviate sepsis-associated acute lung injury. Int. Immunopharmacol..

[33] Wu S., He Y., Li J., Zhuang H., Wang P., He X., Guo Y., Li Z., Shen H., Ye L. (2025). TREM2 alleviates sepsis-induced acute lung injury by attenuating ferroptosis via the SHP1/STAT3 pathway. Free Radic. Biol. Med..

[34] Lin S., Yan J., Wang W., Luo L. (2024). STAT3-Mediated Ferroptosis is Involved in Sepsis-Associated Acute Respiratory Distress Syndrome. Inflammation.

[35] Basisty N., Kale A., Jeon O.H., Kuehnemann C., Payne T., Rao C., Holtz A., Shah S., Sharma V., Ferrucci L. (2020). A proteomic atlas of senescence-associated secretomes for aging biomarker development. PLoS Biol..

[36] Coppé J.P., Desprez P.Y., Krtolica A., Campisi J. (2010). The senescence-associated secretory phenotype: The dark side of tumor suppression. Annu. Rev. Pathol..

[37] Xiang W., Zhang T., Li B., Li S., Zhang B., Fang S., Chen L., Gong Y., Huang B., Feng D. (2025). Senescent macrophages induce ferroptosis in skeletal muscle and accelerate osteoarthri-tis-related muscle atrophy. Nat. Aging.

[38] Huidobro C., Martín-Vicente P., López-Martínez C., Alonso-López I., Amado-Rodríguez L., Crespo I., Albaiceta G.M. (2021). Cellular and molecular features of senescence in acute lung injury. Mech. Ageing Dev..

[39] Wang Y., Zhang H., Miao C. (2025). Unraveling immunosenescence in sepsis: From cellular mechanisms to therapeutics. Cell Death Dis..

[40] Gao L., Zheng F., Fu Z., Wang W. (2026). Ginsenoside Rb1-enhanced decellularized extracellular matrix hydrogels ameliorates mitochondrial dysfunction and cellular aging in sepsis-induced acute lung injury. J. Ginseng Res..

[41] Mansfield L., Ramponi V., Gupta K., Stevenson T., Mathew A.B., Barinda A.J., Herbstein F., Morsli S. (2024). Emerging insights in senescence: Pathways from preclinical models to therapeutic innovations. npj Aging.

[42] Parimon T., Chen P., Stripp B.R., Liang J., Jiang D., Noble P.W., Parks W.C., Yao C. (2023). Senescence of alveolar epithelial progenitor cells: A critical driver of lung fibrosis. Am. J. Physiol. Cell Physiol..

[43] Barnes P.J. (2025). Senotherapy for chronic lung disease. Pharmacol. Rev..

[44] Kourti M., Kontoghiorghes G.J. (2026). Targeting Aging and Diseases Associated with Ferroptosis and Senescence Through Modulation of Iron, Oxidative Stress and Lipid Peroxidation. Antioxidants.

[45] Zhang P., Liu W., Wang S., Wang Y., Han H. (2024). Ferroptosis and Its Role in the Treatment of Sepsis-Related Organ Injury: Mechanisms and Potential Therapeutic Approaches. Infect. Drug Resist..

[46] Dixon S.J., Olzmann J.A. (2024). The cell biology of ferroptosis. Nat. Rev. Mol. Cell Biol..

[47] Galluzzi L., Vitale I., Aaronson S.A., Abrams J.M., Adam D., Agostinis P., Alnemri E.S., Altucci L., Amelio I., Andrews D.W. (2018). Molecular mechanisms of cell death: Recommendations of the Nomenclature Committee on Cell Death 2018. Cell Death Differ..

[48] Yan H.-F., Zou T., Tuo Q.-Z., Xu S., Li H., Belaidi A.A., Lei P. (2021). Ferroptosis: Mechanisms and links with diseases. Signal Transduct. Target. Ther..

[49] Ursini F., Maiorino M. (2020). Lipid peroxidation and ferroptosis: The role of GSH and GPx4. Free Radic. Biol. Med..

[50] Gong F., Zheng X., Xu W., Xie R., Liu W., Pei L., Zhong M., Shi W., Qu H., Mao E. (2025). H3K14la drives endothelial dysfunction in sepsis-induced ARDS by promoting SLC40A1/transferrin-mediated ferroptosis. MedComm.

[51] Fang P., Li S., Lu Z.-Q., Xia D.-L., Xu M.-M., Pan J.-J., Fu L., Sun G.-Y., You Q.-H. (2025). Histone lactylation exacerbates acute lung injury in septic mice by promoting ferroptosis in pulmonary microvascular endothelial cells. Burns Trauma.

[52] Yang W.S., SriRamaratnam R., Welsch M.E., Shimada K., Skouta R., Viswanathan V.S., Cheah J.H., Clemons P.A., Shamji A.F., Clish C.B. (2014). Regulation of ferroptotic cancer cell death by GPX4. Cell.

[53] Friedmann Angeli J.P., Schneider M., Proneth B., Tyurina Y.Y., Tyurin V.A., Hammond V.J., Herbach N., Aichler M., Walch A., Eggenhofer E. (2014). Inactivation of the ferroptosis regulator Gpx4 triggers acute renal failure in mice. Nat. Cell Biol..

[54] Kagan V.E., Mao G., Qu F., Angeli J.P.F., Doll S., Croix C.S., Dar H.H., Liu B., Tyurin V.A., Ritov V.B. (2017). Oxidized arachidonic and adrenic PEs navigate cells to ferroptosis. Nat. Chem. Biol..

[55] Doll S., Proneth B., Tyurina Y.Y., Panzilius E., Kobayashi S., Ingold I., Irmler M., Beckers J., Aichler M., Walch A. (2017). ACSL4 dictates ferroptosis sensitivity by shaping cellular lipid composition. Nat. Chem. Biol..

[56] Gao M., Yi J., Zhu J., Minikes A.M., Monian P., Thompson C.B., Jiang X. (2019). Role of Mitochondria in Ferroptosis. Mol. Cell.

[57] Magtanong L., Mueller G.D., Williams K.J., Billmann M., Chan K., Armenta D.A., Pope L.E., Moffat J., Boone C., Myers C.L. (2022). Context-dependent regulation of ferroptosis sensitivity. Cell Chem. Biol..

[58] Zhang J., Zheng Y., Wang Y., Wang J., Sang A., Song X., Li X. (2022). YAP1 alleviates sepsis-induced acute lung injury via inhibiting ferritinophagy-mediated ferroptosis. Front. Immunol..

[59] Su F., Yan X., Li X., Zeng B., Sun X., Huang C., Chen C., Li S., Chen Y. (2026). SLC38A1 Inhibits Ferroptosis of Alveolar Type II Epithelial Cells in Acute Lung Injury by Promoting Autophagic Degradation of Divalent Metal Transporter 1 (DMT1): An In Vivo and In Vitro Study. Inflammation.

[60] Xiao F., Li D., Yu M., Zhu Y., Huang G., Huang Z., Wang Y., Li J., Zhong D., Ma H. (2026). errostatin-1 protects against early sepsis-induced acute lung injury by suppressing lipid peroxidation–driven NINJ1-mediated DAMP release and neutrophil activation. Redox Biol..

[61] Liu P., Feng Y., Li H., Chen X., Wang G., Xu S., Li Y., Zhao L. (2020). Ferrostatin-1 alleviates lipopolysaccharide-induced acute lung injury via inhibiting ferroptosis. Cell. Mol. Biol. Lett..

[62] Chen K., Lu S., Shi K., Ali M.H., Liu J., Yin F., Yin W. (2025). Hyperoside attenuates sepsis-induced acute lung injury by NRF2 activation and ferroptosis inhibition. Int. Immunopharmacol..

[63] Shimizu J., Murao A., Nofi C., Wang P., Aziz M. (2022). Extracellular CIRP promotes GPX4-mediated ferroptosis in sepsis. Front. Immunol..

[64] Li Z., Yu Y., Bu Y., Liu C., Jin J., Li W., Chen G., Liu E., Zhang Y., Gong W. (2024). QiShenYiQi pills preserve endothelial barrier integrity to mitigate sepsis-induced acute lung in-jury by inhibiting ferroptosis. J. Ethnopharmacol..

[65] Wu D., Spencer C.B., Ortoga L., Zhang H., Miao C. (2024). Histone lactylation-regulated METTL3 promotes ferroptosis via m6A-modification on ACSL4 in sepsis-associated lung injury. Redox Biol..

[66] Feng F., He S., Li X., He J., Luo L. (2024). Mitochondria-mediated Ferroptosis in Diseases Therapy: From Molecular Mechanisms to Implications. Aging Dis..

[67] Zhang J., Wu D., Zeng F., Gu H., Li C., Cata J.P., Guo K., Miao C., Zhang H. (2025). Lactate metabolic reprogramming and histone lactylation modification in sepsis. Int. J. Biol. Sci..

[68] Zeng T., Zhou Y., Yu Y., Wang J.-W., Wu Y., Wang X., Zhu L., Zhou L.-M., Wan L.-H. (2023). rmMANF prevents sepsis-associated lung injury via inhibiting endoplasmic reticulum stress-induced ferroptosis in mice. Int. Immunopharmacol..

[69] Sang A., Zhang J., Zhang M., Xu D., Xuan R., Wang S., Song X., Li X. (2024). METTL4 mediated-N6-methyladenosine promotes acute lung injury by activating ferroptosis in alveolar epithelial cells. Free Radic. Biol. Med..

[70] Deng Y., Qiu Y., Li X., Gong T., Guo J., Liang H., Yuan Z., Hei Z., Zhang X., Liu Y. (2026). PDK4-driven lactate accumulation facilitates LPCAT2 lactylation to exacerbate sepsis-induced acute lung injury. Cell Death Differ..

[71] Li Z., Liu C., Ma Z., Zheng D., Wang R., Yu Y., Chen G., Li C., Bu Y., Cao H. (2026). Targeting the GPX4-FUNDC1 Interaction with Magnesium Lithospermate B Attenuates Sepsis-Associated Lung Injury. Adv. Sci..

[72] Mösenlechner M., Schlösser D., Braumüller S., Dörfer L., Mannes M., Kawach R., Strauss G., Schmidt C.Q., Lupu L., Huber-Lang M.S. (2025). Induction of Early Pulmonary Senescence in Experimental Sepsis. Shock.

[73] Childs B.G., Durik M., Baker D.J., van Deursen J.M. (2015). Cellular senescence in aging and age-related disease: From mechanisms to therapy. Nat. Med..

[74] Gorgoulis V., Adams P.D., Alimonti A., Bennett D.C., Bischof O., Bishop C., Campisi J., Collado M., Evangelou K., Ferbeyre G. (2019). Cellular Senescence: Defining a Path Forward. Cell.

[75] Kumari R., Jat P. (2021). Mechanisms of Cellular Senescence: Cell Cycle Arrest and Senescence Associated Secretory Phenotype. Front. Cell Dev. Biol..

[76] Campisi J., d’Adda di Fagagna F. (2007). Cellular senescence: When bad things happen to good cells. Nat. Rev. Mol. Cell Biol..

[77] van Deursen J.M. (2014). The role of senescent cells in ageing. Nature.

[78] Baker D.J., Childs B.G., Durik M., Wijers M.E., Sieben C.J., Zhong J., Saltness R.A., Jeganathan K.B., Verzosa G.C., Pezeshki A. (2016). Naturally occurring p16Ink4a-positive cells shorten healthy lifespan. Nature.

[79] Kobayashi Y., Tata A., Konkimalla A., Katsura H., Lee R.F., Ou J., Banovich N.E., Kropski J.A., Tata P.R. (2020). Persistence of a regeneration-associated, transitional alveolar epithelial cell state in pulmonary fibrosis. Nat. Cell Biol..

[80] Huang X., Zhang X., Machireddy N., Evans C.E., Trewartha S.D., Hu G., Fang Y., Mutlu G.M., Wu D., Zhao Y.-Y. (2023). Endothelial FoxM1 reactivates aging-impaired endothelial regeneration for vascular repair and resolution of inflammatory lung injury. Sci. Transl. Med..

[81] Huang X., Zhao Y.Y. (2012). Transgenic expression of FoxM1 promotes endothelial repair following lung injury induced by polymicrobial sepsis in mice. PLoS ONE.

[82] Evangelou K., Belogiannis K., Papaspyropoulos A., Petty R., Gorgoulis V.G. (2023). Escape from senescence: Molecular basis and therapeutic ramifications. J. Pathol..

[83] Hirano Y., Ode Y., Ochani M., Wang P., Aziz M. (2018). Targeting junctional adhesion molecule-C ameliorates sepsis-induced acute lung in-jury by decreasing CXCR4+ aged neutrophils. J. Leukoc. Biol..

[84] Biswas N., Bahr A., Howard J., Bonin J.L., Grazda R., MacNamara K.C. (2024). Survivors of polymicrobial sepsis are refractory to G-CSF-induced emergency myelopoiesis and hematopoietic stem and progenitor cell mobilization. Stem Cell Rep..

[85] Cao R., Thatavarty A., King K.Y. (2024). Forged in the fire: Lasting impacts of inflammation on hematopoietic progenitors. Exp. Hematol..

[86] Reyes M., Filbin M.R., Bhattacharyya R.P., Sonny A., Mehta A., Billman K., Kays K.R., Pinilla-Vera M., Benson M.E., Cosimi L.A. (2021). Plasma from patients with bacterial sepsis or severe COVID-19 induces suppressive myeloid cell production from hematopoietic progenitors in vitro. Sci. Transl. Med..

[87] De Zuani M., Lázničková P., Kohoutková M.H., Bosáková V., Andrejčinová I., Vadovičová N., Tomášková V., Mýtniková A., Štíchová J., Tomáš T. (2026). Sepsis induces long-term reprogramming of human HSPCs and drives myeloid dysregulation in sepsis survivors. J. Inflamm..

[88] Kim J.W., Nam S.A., Koh E.-S., Kim H.W., Kim S., Woo J.J., Kim Y.K. (2024). The impairment of endothelial autophagy accelerates renal senescence by ferroptosis and NLRP3 inflammasome signaling pathways with the disruption of endothelial barrier. Antioxidants.

[89] Sun D.-Y., Wu W.-B., Wu J.-J., Shi Y., Xu J.-J., Ouyang S.-X., Chi C., Shi Y., Ji Q.-X., Miao J.-H. (2024). Pro-ferroptotic signaling promotes arterial aging via vascular smooth muscle cell senescence. Nat. Commun..

[90] Feng Y., Wei H., Lyu M., Yu Z., Chen J., Lyu X., Zhuang F. (2024). Iron retardation in lysosomes protects senescent cells from ferroptosis. Aging.

[91] Lv Y., Chen D., Tian X., Xiao J., Xu C., Du L., Li J., Zhou S., Chen Y., Zhuang R. (2023). Protectin conjugates in tissue regeneration 1 alleviates sepsis-induced acute lung injury by inhibiting ferroptosis. J. Transl. Med..

[92] Cao Y., Peng T., Ai C., Li Z., Lei X., Li G., Li T., Wang X., Cai S. (2023). Inhibition of SIRT6 aggravates p53-mediated ferroptosis in acute lung injury in mice. Heliyon.

[93] Tarangelo A., Magtanong L., Bieging-Rolett K.T., Li Y., Ye J., Attardi L.D., Dixon S.J. (2018). p53 Suppresses Metabolic Stress-Induced Ferroptosis in Cancer Cells. Cell Rep..

[94] Shen K., Wang X., Wang Y., Jia Y., Zhang Y., Wang K., Luo L., Cai W., Li J., Li S. (2023). miR-125b-5p in adipose-derived stem cell exosomes alleviates pulmonary microvascular endothelial cell ferroptosis via Keap1/NRF2/GPX4 in sepsis lung injury. Redox Biol..

[95] Luo Y., Long M., Wu X., Zeng L. (2025). Targeting ferroptosis: Novel therapeutic approaches and intervention strategies for kidney diseases. Front. Immunol..

[96] Sinha P., Kerchberger V.E., Willmore A., Chambers J., Zhuo H., Abbott J., Jones C., Wickersham N., Wu N., Neyton L. (2023). Identifying molecular phenotypes in sepsis: An analysis of two prospective observational cohorts and secondary analysis of two randomised controlled trials. Lancet Respir. Med..

[97] Sinha P., Delucchi K.L., McAuley D.F., O’KAne C.M., Matthay M.A., Calfee C.S. (2020). Development and validation of parsimonious algorithms to classify acute respiratory distress syndrome phenotypes: A secondary analysis of randomised controlled trials. Lancet Respir. Med..

[98] Ware L.B., Koyama T., Zhao Z., Janz D.R., Wickersham N., Bernard G.R., May A.K., Calfee C.S., Matthay M.A. (2013). Biomarkers of lung epithelial injury and inflammation distinguish severe sepsis patients with acute respiratory distress syndrome. Crit. Care.

[99] Yang P.M., Iffrig E., Harris F.B., Holder A.L.M., Martin G.S.M., Esper A.M.M. (2022). Serial Measurements of Protein Biomarkers in Sepsis-Induced Acute Respiratory Distress Syndrome. Crit. Care Explor..

[100] Sathe N.A., Morrell E.D.M., Bhatraju P.K.M., Fessler M.B., Stapleton R.D., Wurfel M.M., Mikacenic C. (2023). Alveolar Biomarker Profiles in Subphenotypes of the Acute Respiratory Distress Syndrome. Crit. Care Med..

[101] Delucchi K., Famous K.R., Ware L.B., E Parsons P., Thompson B.T., Calfee C.S. (2018). Stability of ARDS subphenotypes over time in two randomised controlled trials. Thorax.

[102] Doll S., Freitas F.P., Shah R., Aldrovandi M., da Silva M.C., Ingold I., Grocin A.G., da Silva T.N.X., Panzilius E., Scheel C.H. (2019). FSP1 is a glutathione-independent ferroptosis suppressor. Nature.

[103] Bersuker K., Hendricks J.M., Li Z., Magtanong L., Ford B., Tang P.H., Roberts M.A., Tong B., Maimone T.J., Zoncu R. (2019). The CoQ oxidoreductase FSP1 acts parallel to GPX4 to inhibit ferroptosis. Nature.

[104] Zhang T., Dong Y., Li Z., Xia L., Hou C., Liu G., Jin H., Liu Y., Shou S. (2026). STING–FSP1 signaling drives endothelial ferroptosis and vascular leakage in sepsis. Int. Immunopharmacol..

[105] You B., Yang Y., Wei J., Zhou C., Dong S. (2025). Pathogenic and therapeutic roles of extracellular vesicles in sepsis. Front. Immunol..

[106] Tchkonia T., Zhu Y., van Deursen J., Campisi J., Kirkland J.L. (2013). Cellular senescence and the senescent secretory phenotype: Therapeutic opportunities. J. Clin. Invest..

[107] Lavarti R., Cai L., Alvarez-Diaz T., Medina-Rodriguez T., Bombin S., Raju R.P. (2025). Senescence landscape in the liver following sepsis and senolytics as potential therapeutics. Aging Cell.

[108] Silva M., Wacker D.A., Driver B.E., Staugaitis A., Niedernhofer L.J., Schmidt E.L., Kirkland J.L., Tchkonia T., Evans T., Serrano C.H. (2024). STOP-Sepsis Investigators. Senolytics To slOw Progression of Sepsis (STOP-Sepsis) in elderly patients: Study protocol for a multicenter, randomized, adaptive allocation clinical trial. Trials.

[109] Wang T., Zhang Z., Deng Z., Zeng W., Gao Y., Hei Z., Yuan D. (2024). Mesenchymal stem cells alleviate sepsis-induced acute lung injury by blocking neutrophil extracellular traps formation and inhibiting ferroptosis in rats. PeerJ.

[110] Bai X., Liu Y., Liu J., Guo K., Guan H. (2025). ADSCs-derived exosomes suppress macrophage ferroptosis via the SIRT1/NRF2 signaling axis to alleviate acute lung injury in sepsis. Int. Immunopharmacol..

[111] Matthay M.A., Calfee C.S., Zhuo H., Thompson B.T., Wilson J.G., E Levitt J., Rogers A.J., E Gotts J., Wiener-Kronish J.P., Bajwa E.K. (2019). Treatment with allogeneic mesenchymal stromal cells for moderate to severe acute respiratory distress syndrome (START study): A randomised phase 2a safety trial. Lancet Respir. Med..

[112] Gorman E., Shankar-Hari M., Hopkins P., Tunnicliffe W.S., Perkins G.D., Silversides J., McGuigan P., Krasnodembskaya A., Jackson C., Boyle R. (2021). Repair of acute respiratory distress syndrome by stromal cell administration (REALIST) trial: A phase 1 trial. EClinicalMedicine.

[113] Wick K.D., Leligdowicz A., Zhuo H., Ware L.B., Matthay M.A. (2021). Mesenchymal stromal cells reduce evidence of lung injury in patients with ARDS. JCI Insight.

[114] Kastrup J., Haack-Sørensen M., Juhl M., Søndergaard R.H., Follin B., Lund L.D., Johansen E.M., Qayyum A.A., Mathiasen A.B., Jørgensen E. (2017). Cryopreserved Off-the-Shelf Allogeneic Adipose-Derived Stromal Cells for Therapy in Patients with Ischemic Heart Disease and Heart Failure—A Safety Study. Stem Cells Transl. Med..

[115] Hosseinzadeh M., Pouladzadeh M., Khedri R., Oskouei S.Y., Amini P., Dargahi-Malamir M., Kamyari N., Hosseininejad H., Seyedian S.S., Shoae-Hassani A. (2025). Therapeutic potential of hUCMSC-derived exosomes in modulating inflammation and immune response in COVID-19-associated ARDS: A randomized clinical trial. Respir. Med..

[116] He D., Yu Q., Zeng X., Feng J., Yang R., Wan H., Zhong Y., Yang Y., Zhao R., Lu J. (2023). Single-cell RNA sequencing and transcriptome analysis revealed the immune microenvironment and gene markers of acute respiratory distress syndrome. J. Inflamm. Res..

[117] Flerlage T., Crawford J.C., Allen E.K., Severns D., Tan S., Surman S., Ridout G., Novak T., Randolph A., West A.N. (2023). Single cell transcriptomics identifies distinct profiles in pediatric acute respiratory distress syndrome. Nat. Commun..

[118] Yang R., Zheng T., Xiang H., Liu M., Hu K. (2024). Lung single-cell RNA profiling reveals response of pulmonary capillary to sepsis-induced acute lung injury. Front. Immunol..

[119] Niethamer T.K., Planer J.D., Morley M.P., Babu A., Zhao G., Basil M.C., Cantu E., Frank D.B., Diamond J.M., Nottingham A.N. (2025). Longitudinal single-cell profiles of lung regeneration after viral infection reveal persistent injury-associated cell states. Cell Stem Cell.

[120] Barkauskas C.E., Cronce M.J., Rackley C.R., Bowie E.J., Keene D.R., Stripp B.R., Randell S.H., Noble P.W., Hogan B.L.M. (2013). Type 2 alveolar cells are stem cells in adult lung. J. Clin. Investig..

[121] Huang L.S., Hong Z., Wu W., Xiong S., Zhong M., Gao X., Rehman J., Malik A.B. (2020). mtDNA activates cGAS signaling and suppresses the YAP-mediated endothelial cell proliferation program to promote inflammatory injury. Immunity.

[122] Zhu J., Zhou J., Feng B., Pan Q., Yang J., Lang G., Shang D., Zhou J., Li L., Yu J. (2024). MSCs alleviate LPS-induced acute lung injury by inhibiting the proinflammatory function of macrophages in mouse lung organoid–macrophage model. Cell. Mol. Life Sci..

[123] Zhang M., Wang P., Luo R., Wang Y., Li Z., Guo Y., Yao Y., Li M., Tao T., Chen W. (2021). Biomimetic human disease model of SARS-CoV-2-induced lung injury and immune responses on organ chip system. Adv. Sci..

[124] van der Koog L., Wu X., Nugraha D.F., Ravi A., Wolters J.C., Verhamme F.M., Horvatovich P.L., Bracke K.R., van der Does A.M., Hiemstra P.S. (2025). Pulmonary microvascular endothelial cells support alveolar epithelial growth via bone morphogenetic protein 6 secretion. Am. J. Respir. Cell Mol. Biol..

